# Transmission-selective muscle pathology induced by the active propagation of mutant huntingtin across the human neuromuscular synapse

**DOI:** 10.3389/fnmol.2023.1287510

**Published:** 2024-01-03

**Authors:** Margarita C. Dinamarca, Laura Colombo, Urszula Brykczynska, Amandine Grimm, Isabelle Fruh, Imtiaz Hossain, Daniela Gabriel, Anne Eckert, Matthias Müller, Eline Pecho-Vrieseling

**Affiliations:** ^1^Neuronal Development and Degeneration Laboratory, Department of Biomedicine, University of Basel, Basel, Switzerland; ^2^Neurobiology Laboratory for Brain Aging and Mental Health, Transfaculty Research Platform, Molecular and Cognitive Neuroscience, University of Basel, Basel, Switzerland; ^3^Biomedical Research, Novartis Pharma AG, Novartis Campus, Basel, Switzerland

**Keywords:** hiPSC, neuromuscular system, synaptic, protein transmission, neurodegeneration, mutant huntingtin, Huntington's disease

## Abstract

Neuron-to-neuron transmission of aggregation-prone, misfolded proteins may potentially explain the spatiotemporal accumulation of pathological lesions in the brains of patients with neurodegenerative protein-misfolding diseases (PMDs). However, little is known about protein transmission from the central nervous system to the periphery, or how this propagation contributes to PMD pathology. To deepen our understanding of these processes, we established two functional neuromuscular systems derived from human iPSCs. One was suitable for long-term high-throughput live-cell imaging and the other was adapted to a microfluidic system assuring that connectivity between motor neurons and muscle cells was restricted to the neuromuscular junction. We show that the Huntington's disease (HD)-associated mutant HTT exon 1 protein (mHTTEx1) is transmitted from neurons to muscle cells across the human neuromuscular junction. We found that transmission is an active and dynamic process that starts before aggregate formation and is regulated by synaptic activity. We further found that transmitted mHTTEx1 causes HD-relevant pathology at both molecular and functional levels in human muscle cells, even in the presence of the ubiquitous expression of mHTTEx1. In conclusion, we have uncovered a causal link between mHTTEx1 synaptic transmission and HD pathology, highlighting the therapeutic potential of blocking toxic protein transmission in PMDs.

## 1 Introduction

Neurodegenerative protein-misfolding diseases (PMDs) comprise a group of unrelated illnesses that include Alzheimer's disease (AD), Parkinson's disease (PD), Huntington's disease (HD), amyotrophic lateral sclerosis (ALS), and frontotemporal lobar dementia (FTLD). These disorders are all characterized by the misfolding and aggregation of disease-specific proteins, cell type-specific vulnerability to degeneration, and the progressive loss of nervous system structure and function. Disease processes have already been active for many years before symptoms become apparent, usually around middle age. They initially present as discrete neurobehavioral and neuropsychiatric manifestations that progressively worsen into cognitive impairment (Schmahmann et al., 2008). Currently, no therapies are available to cure or slow the progression of these devastating illnesses.

It has been suggested that neuron-to-neuron transmission of toxic, misfolded protein species may potentially explain the spatiotemporal propagation of pathological lesions through the brains of patients with PMDs (Brundin et al., 2010; Vaquer-Alicea and Diamond, 2019; Peng et al., 2020). A causal link between the transcellular spreading of misfolded prion proteins [PrP scrapie (PrPSc)] and pathology has been demonstrated in prion diseases (Prusiner, 1982, 2013). Regarding other neurodegenerative PMDs, it has been firmly demonstrated that tau (AD), alpha-synuclein (α-syn) (PD), mutant huntingtin (mHTT) (HD), and TDP-43 (ALS and FTLD) are transmitted between cells as well as between functionally connected brain regions [reviewed in Guo and Lee (2014); Vaquer-Alicea and Diamond (2019); Alpaugh et al. (2021)]. This transmission is accompanied by the appearance of protein aggregates in acceptor cells. Furthermore, a decline in cognitive and motor behavior has been associated with region- or cell type-specific misfolded protein expression (Liu et al., 2012; Luk et al., 2012a,b; Jeon et al., 2016; Masnata et al., 2019).

Several cellular mechanisms have been proposed to underlie the inter-neuronal transmission of misfolded proteins (Masnata and Cicchetti, 2017; Brunello et al., 2020; Donnelly et al., 2020; Peng et al., 2020). It has been shown that the transneuronal transmission of Aβ, tau, and mHTT is regulated by neuronal activity and synaptic connectivity (Cirrito et al., 2005; Pecho-Vrieseling et al., 2014; Babcock and Ganetzky, 2015; Calafate et al., 2015; Wang et al., 2017; Donnelly et al., 2020). This, together with the observations that tau, α-syn, mHTT, and TDP-43 are transmitted between functionally connected brain regions *in vivo* in both mice and *Drosophila*, strongly suggests the existence of a transsynaptic transmission pathway for misfolded proteins (Braak and Del Tredici, 2011; de Calignon et al., 2012; Liu et al., 2012; Luk et al., 2012a,b; Rey et al., 2013; Pecho-Vrieseling et al., 2014; Babcock and Ganetzky, 2015; Jeon et al., 2016). Synaptic connections also connect the central nervous system (CNS) with the periphery, such as the neuromuscular synapsis, referred to as neuromuscular junctions (NMJs), that form between spinal motor neurons and skeletal muscles. In *Caenorhabditis elegans*, mHTT has been shown to translocate bidirectionally between the CNS and skeletal muscles (Kim et al., 2017), implying that the transmission of misfolded proteins may represent a systemic disease pathway that not only affects the CNS but also contributes to the progressive deterioration of peripheral systems.

Patients with HD exhibit a progressive decline in skeletal muscle function (Turner et al., 2007; Zielonka et al., 2014). HD is an autosomal dominant disorder that has 100% penetrance when the number of CAG triplet repeats in exon 1 of the *HTT* gene equals or exceeds 40 repeats. This repeat is translated into a pathogenic polyglutamine stretch in the HTT protein (The Huntington's Disease Collaborative Research Group, 1993). Incomplete *HTT* mRNA splicing results in a toxic exon 1 fragment of the protein (HTTEx1) that is highly prone to aggregation and aberrantly translocates to the nucleus, where it interferes with transcription (Saudou et al., 1998; Ross and Tabrizi, 2011; Neueder et al., 2018; Yang et al., 2020). Human neuronal cell lines overexpressing HTTEx1 develop intra-nuclear inclusions and mitochondrial dysfunction (Ghosh et al., 2020). These pathologies are also observed in the skeletal muscles of patients with HD and animal models of the disease, together with skeletal muscle wasting and fatigue (Lodi et al., 2000; Orth et al., 2003; Ciammola et al., 2006, 2011; Zielonka et al., 2014; Kojer et al., 2019; Bozzi and Sciandra, 2020).

Using an isogenic human-induced pluripotent stem cell (hiPSC) neuromuscular model combined with high-throughput live-cell imaging, functional analysis, and microfluidic systems, we addressed whether neuron-to-muscle transmission of mHTTEx1 can occur across the NMJ and how synaptic density and activity can influence this process. We also examined whether the neuromuscular transmission of mHTT can contribute to skeletal muscle pathology. We show that mHTTEx1 is transmitted from neurons to myotubes across the NMJ and that transmission is elevated by increased NMJ density and modulated by neuronal activity. Moreover, our data revealed that transmission occurs independent of mHTTEx1 aggregation and is enhanced during NMJ functional maturation. Our data further showed that mHTTEx1 transmission results in fragmented mitochondria, an increase in the number of intra-nuclear aggregates, and a decline in myotube contractibility. Importantly, these pathologies were enhanced or specifically induced by transmission in the presence of cell-autonomous mHTTEx1 in myotubes. Our findings suggest that cell-to-cell transmission of mHTTEx1 occurs between the CNS and the periphery and might contribute to pathological alterations in the neuromuscular system at early, preclinical stages of the disease. More broadly, our findings also support the fact that cell type-specific vulnerability might be determined by the level of functional synaptic connectivity in combination with the transsynaptic transmission of misfolded proteins.

## 2 Materials and methods

### 2.1 iPSC culture and characterization

The hiPSCs used in this study were generated as previously described (Russell et al., 2018) from the human dermal fibroblast line C-013-5C (Thermo Fisher) derived from a healthy 32-year-old female. The hiPSCs were cultured on Matrigel-coated dishes using mTeSR 1 medium supplemented with 1% penicillin/streptomycin (Thermo Fisher, 15070-063). The pluripotency of the cells was assessed by FACS analyses using the Human Pluripotent Stem Cell Sorting and Analysis Kit (BD Biosciences, 560461), following the protocol provided by the manufacturer. SSEA-3 and TRA-1-81 were used as pluripotency markers, and SSEA-1 was employed as a differentiation marker. The expression of specific pluripotency markers was also analyzed using conventional immunocytochemistry, as follows: Cells were fixed in 4% paraformaldehyde (PFA) for 15 min at room temperature (RT), permeabilized with 0.1% Triton X-100 for 5 min, also at RT, blocked in 1% bovine serum albumin for 1 h at RT, and incubated overnight at 4°C with primary antibodies targeting SSEA-4 (1:1,000; Stemgent, 09-0006), Nanog (1:200; Cell Signaling Technology, #4893), TRA-1-81 (1:200; Stemgent, 09-0011), and OCT4 (1:200; Stemgent, 09-0023). After washing, the cells were incubated with the corresponding secondary antibodies [goat anti-mouse IgG, 1:500 (Invitrogen, T6390) or goat anti-rabbit IgG, 1:500 (Invitrogen, A11008)]. Nuclei were counterstained with 0.2 μg/ml DAPI for 20 min at RT, and images were captured with a Carl Zeiss imaging system using the AxioVision software (Carl Zeiss). The differentiation potential of the cells was tested using the hPSC Scorecard Assay (Thermo Fisher, A15876) following the manufacturer's protocol, and the data were analyzed using the ScoreCard Analysis cloud-based software (Thermo Fisher). The karyotype of the cells was assessed by whole-genome SNP analysis [Life&Brain Gmbh (Bonn)], and all lines showed a normal karyotype.

### 2.2 Generation and differentiation of *Neu* hiPSCs to iND3 neurons

The neuronal differentiation protocol was as described in Russell et al. (2018), with small modifications. Briefly, hiPSCs were plated on Matrigel in proliferation medium [DMEM/F12 with GlutaMAX (Gibco, 10565-018), 2% B27 (Thermo Fisher, 17504-044), and 1% N2 (Thermo Fisher, 17502-048) supplements; 1% penicillin/streptomycin (Thermo Fisher, 15070-063); 10 ng/ml hEGF (Thermo Fisher, PHG0315); 10 ng/ml hFGF (Invitrogen, CTP0263), and 10 μM ROCK inhibitor (RI)] for 1 day and 1 μg/ml doxycycline (DOX) for 3 days. Progenitor cells were kept frozen in CryoStor freezing medium (STEMCELL Technology, 07930) or immediately replated for experiments.

### 2.3 Generation of *MyoD* hiPSCs and differentiation to iMD3 myoblasts

Human myoblast determination protein 1 (MyoD) cDNA was synthesized based on sequence information in the Ensembl database (Accession number: NM_002478) and cloned under the control of the TRE (Tetracycline Response Element) tight promoter into a PiggyBac (PB)/Tet-ON all-in-one vector (Lacoste et al., 2009). This vector contains a CAG rtTA16 cassette that allows the constitutive expression of the Tet-ON system and an Hsv-tkNeo cassette for the generation of stable iPSC clones. *MyoD* hiPSCs were generated following a previously described protocol (Russell et al., 2018). Briefly, 1 × 10^6^ hiPSCs were nucleofected with 4 μg of MyoD plasmid and 1 μg of the dual helper plasmid in an Amaxa nucleofector device (program B-016) using Human Stem Cell Nucleofector Kit 1 (Lonza, VPH-5012). Subsequently, the cells were replated on Matrigel plates with NutriStem medium containing 10 μM RI. Antibiotic (geneticin) selection (G418, 0.1 mg/ml) was performed after 48 h, and stable clones appeared within 1 week.

*MyoD* hiPSCs were seeded on plates coated with 5 μg/ml laminin 521 (Biolamina) in alpha MEM (Gibco, 12571-063) containing 5% KSR (Gibco, 10828028), 1% penicillin/streptomycin (Gibco, 15140-122), 100 μM β-mercaptoethanol (Gibco, 21985-023), 1 μg/ml DOX, and 10 μM RI for 1 day. After 24 h, the medium was changed to 5% KSR medium containing 1 μg/ml DOX. After 3 days of seeding, the cells [named iMD3 (hiPSC-derived myoblasts Day 3); Supplementary Figure 2A] were frozen in CryoStor freezing medium or replated.

### 2.4 Plasmid construction

Pathological human huntingtin exon1 carrying 72 glutamines was fused to the Cre recombinase sequence (HTTEx1Q72-Cre) or mCherry (HTTEx1Q72-mCherry) under the control of the CAG promoter in a PB plasmid. A second PB plasmid was designed to carry a lox-stop-lox_GFP sequence (driven by the same CAG promoter). The HTTEx1Q72-Cre, HTTEx1Q72-mCherry, and lox-stop-lox_GFP constructs were synthesized and cloned into the PB backbone by Life Technology Europe BV. The three PB plasmids were subsequently nucleofected into neurogenin 2 (*NGN2*) hiPSCs or *MyoD* hiPSCs.

### 2.5 Generation of stable Cre-, mCherry-, and LoxP-expressing lines

A single-cell suspension of hiPSCs was collected following detachment (5 min at 37°C) with TrypLE Express Enzyme (Gibco, 12604-013). A total of 1 × 10^6^ cells were resuspended in 100 μl of nucleofection hESC solution 1 (Human Stem Cell Nucleofector Kit 1; Lonza, #VPH-5012) already containing 4 μg of PB construct and 1 μg of the dual helper plasmid (expressing transposase). Nucleofection was performed using program B-016 of the Amaxa nucleofector II device. Immediately after transfection, the cells were seeded in 6-cm Matrigel-coated dishes containing mTeSR-1 medium supplemented with 10 μM RI. Puromycin (1 μg/ml) selection was started after 48–72 h. Clones were picked after 10 days and were seeded in a new 35-mm Matrigel-coated dish for amplification of the new stable lines.

The stability of the lines was tested by the temporal transfection of the Q72-Cre construct into the LoxP-GFP cell line and *vice versa*. Fluorescence was monitored daily under an EVOS microscope to check their functionality. In parallel, the presence of HTTEx1Q72-Cre or HTTEx1Q72-mCherry was checked via western blot using the MAB5492 anti-HTT antibody (1:5,000; Sigma-Aldrich).

### 2.6 From iMD3 to neuromuscular on-top co-culture for live imaging

iMD3 cells were replated or thawed and seeded (2.5 × 10^6^ cells per dish) on 10-cm laminin 521-coated dishes with 5% KSR medium containing 20 ng/ml hFGF (Invitrogen, CTP0263) and 10 μM RI. The medium was replaced with RI-free medium after 24 h. After 3 days (when the cells reached confluence), the cells were detached with TrypLE Express Enzyme (Gibco, 12604-013), counted, and seeded in medium C (DMEM/F12-GlutaMAX, 5% FBS (HyClone, SH30070.02), 0.35% BSA (Sigma, A1595), 1% penicillin/streptomycin + ITS (insulin; 5 μg/ml, transferrin; 5 μg/ml, selenious acid; 5 ng/ml) 1:500 (354351, BD), 2 μM CHIR99021 (1046, Sigma), 1 μM dorsomorphin (Stemgent, 04_0024), 1 mM dibutyryl-cAMP (Biotrend, BS0062), 1 μg/ml DOX, and 10 μM RI. A total of 2 × 10^5^ cells per well were seeded on laminin 521-coated wells of 96-well IBIDI μ-plates (IBIDI, 89626). RI and DOX were removed after 1 day. The medium was changed every other day until day 7 after seeding.

After thawing, iND3 was seeded over myotube cultures. A total of 1.8 × 10^5^ iND3 were plated in neuronal differentiation medium composed of Neurobasal Medium (Thermo Fisher, 21103049); B27 supplement with vitamin A (Invitrogen, 17504-044); N2 supplement (17502-048, Invitrogen); 1% penicillin/streptomycin/GlutaMAX; and BDNF, GDNF, and hNT3 (all at 10 ng/ml; all from R&D). From day 2 of co-culture (DCC 2), the medium was changed every other day.

Live-cell imaging (37°C, 5% CO_2_) of neuromuscular co-cultures was performed on days 4, 7, 14, 21, and 28 with Operetta (Perkin Elmer) using a 10 × (NA 0.4) objective. GFP-positive cells were counted manually.

### 2.7 Calcium imaging in myotubes

Intracellular Ca^2+^ measurement was performed with the Functional Drug Screening System (FDSS) using 384-well Hamamatsu microplates. Cells were transfected with pGP-CMV-GCaMP6f, a genetically encoded calcium indicator that responds to Ca^2+^ in the cytosol (Addgene, plasmid #40755). In the presence of calcium, cells become GFP-positive. The Ca^2+^ Assay Buffer contained 50 ml of 10 × HBSS (Invitrogen, 14065049), 10 ml of HEPES, 1 M (Invitrogen, 15630056), 375 μl of 1 M CaCl_2_, and distilled water to 500 ml. The pH was adjusted to 7.4 with 5 M NaOH, and the buffer was stored at 4°C for 1–2 weeks. AMPA (Sigma, A6816; 10 mM stock, dissolved in H_2_O, stored at −20°C; working concentration: 3 μM, diluted in Ca^2+^ assay buffer) and glutamate (Sigma, 49621; freshly prepared in Ca^2+^ assay buffer) were used to induce Ca^2+^ flux. After diluting in the Ca^2+^ assay buffer, AMPA and glutamate were transferred to a 384-well polypropylene V-bottom plate (Greiner, 781281). The concentration of each compound was adjusted to the volume of liquid on top of the cells. Bungarotoxin (BgTx) (Tocris, 21-331; 1 mM stock in H_2_O) was used to block the acetylcholine receptors (AChRs) on the myotubes. The procedure was as follows: 2 h before measurement, BgTx (5 μg/ml) was added to the culture medium, the cells were washed three times with Ca^2+^ buffer ± BgTx, incubated for 40 min, and then transferred to the FDSS. The FDSS settings were as follows: Fluo3 (EMCCD), Long Expo: 500 ms, injection interval: 0.53 s, total measurements: 300, injection: after 50 measurements (10 μl/well to get a final concentration of 3 μM AMPA or 100 μM glutamate). After measurement, the samples were washed twice with PBS and twice with differentiation medium. After 24 h, a second measurement was performed. The samples were then washed twice with pre-warmed Ca^2+^ buffer, 60 μl/well, followed by the addition of pre-warmed Ca^2+^ buffer (40 μl/well, 37°C) and incubation at 37°C with 5% CO_2_ for 40 min. Measurements were then made in the μCELL/FDSS machine using the same setting as on the previous day. Sensitivity was adjusted to the plate background. For data analysis, the baseline fluorescence was calculated as the average fluorescence before injection. Peak fluorescence was calculated as the maximum fluorescence after injection of the compound minus the baseline fluorescence. The unit Y served as the arbitrary unit of fluorescence intensity.

### 2.8 Cell culture inserts

iND3 and iMD3 were seeded in Culture-Insert 2 wells (IBIDI, 81176) in separate areas of the wells at the appropriate densities. iND3 was seeded on poly-L-lysine (Sigma, P1524) and laminin 521, while iMD3 was seeded on laminin 521. The cells were monitored using an EVOS M7000 microscope on days 1, 4, 8, and 15 after the removal of the insert to check for the presence of GFP-positive cells.

### 2.9 Contractility assay in on-top co-culture

The primary readout for the amount of contraction in any on-top co-culture was captured as the total amount of motion within any given field of view over time using a Yokogawa CV7000 microscope with a 10 × objective (NA 0.3). The raw images (60 per field, 4 fields of view per well) were acquired as a series of 2,560 × 2,180, 16-bit, gray-scale bright-field images at a frequency of 2 Hz. The assay was performed under live cell-imaging conditions (37°C, 5% CO_2_). At least three wells were sampled per experimental condition.

For each consecutive pair of image frames, a motion field was computed, which provided, for each pixel location, a direction and magnitude of projected spatial motion. Thus, for *N* image frames, *N*−1 motion frames were obtained. A numerical threshold for the magnitude of the motion vectors was applied to eliminate possible noise and vibration artifacts and obtain a reliable binary image map of the region-of-contraction. The union of all such pixels over all motion frames in the time series was computed and used as the final region-of-contraction map for comparative analysis between cell lines or treatments. These values were used to describe the on-top co-culture functionality as follows:

- Total contracting area normalized to the well area (%): sum of moving pixels normalized to the area of the acquired fields (namely, the sum of the pixels occupying the four fields of view).
- Active images per well-normalized to the total number of images (%), where active images are images in which pixel movement was detected.

### 2.10 Antibodies, dyes, and other reagents

The following antibodies were used for western blot: anti-MHC3 (embryonic myosin) (1:500; F1.652, DHSB), anti-MHC8 (postnatal myosin) (1:500; DSHB, N3.36), anti-islet 1 (ISL1) (1:1,000; R&D, AF1837-SP), anti-doublecortin (DCX) (1:2,000; Cell Signaling Technology, 4604), anti-choline acetyltransferase (ChAT) (1:1,000, Merck, AB144P), anti-OCT4 (1:250; Stemgent, 09-0023), anti-GAPDH (1:5,000; Abcam, ab9485), anti-β-actin (1:5,000; Sigma, A5441), and anti-HTT (1:5,000; Sigma-Aldrich, MAB5492).

The following reagents were used for immunofluorescence assays: Hoechst 33342, BgTx (5 μg/ml; Thermo Fisher, B1196), anti-bassoon antibody (1:2,000; Synaptic Systems, 141 013), tetanus neurotoxin (TeNT) (2 μg/ml; Sigma, T3194-25UG), anti-neurofilament M antibody (1:1,000; Synaptic Systems, 171 204), anti-myosin heavy chain 1 (MHC 1) antibody (1:1,000; Millipore, 05-716), anti-mCherry antibody (1:5,000, Abcam, ab205402), anti-TOMM20 antibody (1:500; Abcam, ab186735), anti-HTT antibody (1:500; Merck, MAB5374, clone mEM48), anti-ChAT antibody (1:100; Merck, AB144P), anti-MAP2 antibody (1:2,000; Abcam, ab5392), anti-V5 antibody (1:800; Cell Signaling Technology, D3H8Q), and anti-NF2H3 antibody (1:40; DSHB, AB2314897). All the secondary antibodies were Alexa-conjugated and were obtained from Jackson ImmunoResearch. They were all used at 1:1,000 dilutions for 1 h at RT.

### 2.11 Immunocytochemistry for GFP+ cells

Cells were fixed in 4% PFA at RT for 7 min, washed three times in DPBS (Sigma-Aldrich, 14190), 5 min each wash, and incubated with primary antibodies diluted in DPBS supplemented with 0.1% Triton X-100 (for permeabilization) and 1% BSA (blocking) overnight at 4°C. After three washes with DPBS, the cells were incubated with secondary antibodies (Invitrogen) for 1 h at RT, washed again with DPBS, and incubated with Hoechst 33342 in ddH_2_O for 10 min. Images were acquired with an LSM900 microscope using a Plan-Apochromat 63x/1.40 Oil DIC M27 objective and the Zen 3.2 (Blue edition) software.

### 2.12 Western blot

IPSC-derived neurons, myotubes, and co-cultured cells were harvested at different time points, washed twice with ice-cold PBS, and subsequently lysed in RIPA buffer supplemented with a complete EDTA-free protease inhibitor mixture (Roche, 11873580001). The lysates were incubated on ice for 15 min and centrifuged at 10,000 × *g* for 10 min at 4°C. The protein concentration in the supernatant was determined using a BCA assay kit (Thermo Scientific Pierce, 23227). The proteins were subsequently resolved by standard SDS-PAGE, blocked, and incubated with primary antibodies overnight at 4°C. After washing, the samples were incubated with secondary antibodies and visualized using a SuperSignal Femto chemiluminescence detection kit (Thermo Scientific, 35080) in an Odyssey Infrared Imager (LiCor, 9120).

### 2.13 Immunofluorescence in coverslip cultures

Cells seeded on glass coverslips in 24-well plates (3 × 10^5^ myotubes and 3 × 10^5^ neurons per well) were fixed for 5 min in 4% PFA/4% sucrose at RT, permeabilized with PBS+/+ (Sigma, D8662; PBS supplemented with 1 mM MgCl_2_ and 0.1 mM CaCl_2_) containing 0.1% Triton X-100, blocked with 5% BSA in PBS+/+, and labeled with primary antibodies diluted in PBS+/+ (Sigma, D8662) and 5% BSA overnight at 4°C. After washing with PBS+/+, the cells were incubated with secondary antibodies for 1 h at RT. The coverslips were mounted on glass slides in ProLong Gold Antifade (Invitrogen, P36930).

### 2.14 Microfluidic device culture

Xona Chips XC450 devices from Xona Microfluidics were used for MFD culture. iMD3 was grown in alpha-MEM (Gibco, 12571-063) supplemented with 5% KSR (Gibco, 10828028), 1% penicillin/streptomycin (Gibco, 15140-122), 100 μM β-mercaptoethanol (Gibco, 21985-023), 1 μg/ml DOX (Sigma, D1822), and 20 ng/ml FGF (Gemini Bio, 300-112P). On day *in vitro* (DIV) 3, the cells were seeded for differentiation. A total of 3 × 10^5^ cells were seeded on the myocyte side and 1.5 × 10^5^ cells were seeded on the neuronal side in 5 μl of medium to provide support for the motor neurons. Myotubes were allowed to grow for 7 days in differentiation medium [DMEM F12-GlutaMAX (Gibco, 10565-018), 5% FBS (HyClone, SH30070.02), ITS (1:500; BD, 354351), 0.1% BSA (Sigma, A1595), 1% penicillin/streptomycin, 2 μM CHIR99021 (Sigma, 1046), 1 μM dorsomorphin (Stemgent, 04_0024), and 1 mM dibutyryl-cAMP (Biotrend, BS0062)]. Subsequently, 3 × 10^5^ neurons were seeded in the neuronal compartment and grown in neuronal medium [Neurobasal Medium; B27 supplement (Invitrogen, 17504-044); N2 supplement (Invitrogen, 17502-048); 1% penicillin/streptomycin/GlutaMAX; and BDNF, GDNF, and hNT3 (all from R&D)].

For immunofluorescence experiments, the culture was fixed at different time points for 10 min in 4% PFA/4% sucrose at RT, followed by immunofluorescence analysis, as described above.

### 2.15 Mitochondrial morphology quantification

Mitochondrial shape parameters were assessed using ImageJ as previously described [Measuring Mitochondrial Shape with ImageJ. In: Strack S., Usachev Y. (eds) Techniques to Investigate Mitochondrial Function in Neurons. Neuromethods, vol. 123. Humana Press, New York, NY. https://doi.org/10.1007/978-1-4939-6890-9_2]. Briefly, images were subjected to background subtraction (rolling ball radius = 50 pixels), and uneven labeling of mitochondria was improved through local contrast enhancement using contrast-limited adaptive histogram equalization (“CLAHE”). To segment mitochondria, the “Tubeness” filter was applied. After setting an automated threshold, the “Analyze Particles” plugin was used to determine the area and perimeter of individual mitochondria, and the “Skeletonize” function was used to measure mitochondrial length. The following three parameters were assessed: (1) Mitochondrial length, reported as the mitochondrial length or elongation in pixels after the mitochondria were reduced to a single-pixel-wide shape (“Skeletonize” function in ImageJ). (2) Form factor (FF): the FF value describes the mitochondrial particle shape complexity as the inverse of the circularity of individual mitochondria. (3) Area-weighted form factor (AWFF): a variant of FF with a bias toward larger mitochondria or mitochondrial networks. The AWFF provides more realistic results in cases where highly elongated mitochondria overlap.

### 2.16 Image acquisition and analysis

Fluorescence signals in “on-top” cultures of iPSC-derived co-cultures were imaged with the Zeiss LSM-700 system using a Plan-Apochromat 40 × NA 1.30 oil DIC objective and the Zen 2010 software. For bin analysis in the MFD culture experiment, 0–160-, 160–320-, and 320–480-μm sections were imaged with the Zeiss LSM-800II inverted system employing a Plan-Apochromat 40 × NA 1.30 oil DIC objective using the Zen Blue 2.6 software. Whole-cell, 16-bit image stacks with a 0.33-μm step size were acquired (15–30 planes). Immersion oil with a 1.518 refractive index at RT was applied to the lens. Coverslips were mounted with ProLong Gold Antifade reagent (Thermo Fisher, P36930) with a refractive index of 1.46. All images were acquired with identical microscope settings within individual experiments. Brightness and contrast were equally adjusted for all the images, and cropped insets were generated in the same manner among all the experiments to facilitate the visualization of representative cells. Saturation was avoided by using the image acquisition software to monitor intensity values. For any image adjustment, identical settings were always applied to all cells, irrespective of genotype. Cells that were clumped or overlapping were excluded from quantification. For quantification, values were averaged over multiple cells from at least three independent culture preparations.

The number and volume of HTTEx1Q72-mCherry puncta were quantified using the Imaris software (v.9.6.0; Oxford Instruments), and measurement was based on mCherry fluorescence staining. Aggregates with a volume >0.02 and smaller than 30 μm^3^ and localized within the surface generated based on MHC1 staining were analyzed.

Intracellular aggregate localization was analyzed using distances between surfaces generated by the Imaris software based on mCherry staining for aggregates and MHC1 staining for muscle. Localization at the surface was defined as a distance between 0 and 0.05 μm.

Nuclei containing HTTEx1Q72-mCherry were quantified using the ImageJ software. Images were background-subtracted, and, after setting an automated threshold, a mask for DAPI-positive nuclei (in MHC1-positive myotubes or MAP2-positive neurons) was applied to the images, and the “Analyze Particles” plugin was used to determine the number of puncta per nucleus.

Integrated density measurement of mEM48/MAP2+NF staining was done using the ImageJ software. Images were subjected to background subtraction, and, after setting an automated threshold, the integrated density was measured in the whole image, considering mEM48 staining in the soma and neurites of the neurons. The values were normalized to the neuronal markers MAP2 and NF.

AChR clusters were analyzed using the Imaris software (v.9.6.0; Oxford Instruments) based on immunofluorescence images acquired by confocal microscopy. 3D reconstruction of AChR and BSN structures was performed using the Imaris Surface function. Automatically generated values for volume and sphericity were used to characterize the clusters. Only structures with a volume larger than 0.02 and smaller than 20 μm^3^ were analyzed for BSN, and only structures with a volume larger than 0.024 μm^3^ were analyzed for AChR. Distances between surfaces provided by the Imaris software were used to identify AChR cluster-BSN associations. An association was defined as a distance below 0.05 μm.

Multiple images were analyzed using the Imaris Batch function. Data related to volume, sphericity, and distances between surfaces were exported and further analyzed in R (v.4.0.5; https://www.R-project.org/) and RStudio (v.1.4.1106, https://www.rstudio.com/) using base R and ggplot2 (v.3.3.5).

### 2.17 Statistical analysis

Data analysis was performed with GraphPad Prism version 8.0 (GraphPad Software, La Jolla, CA, USA) and the R software (v.4.0.5; https://www.R-project.org/). Individual data sets were tested for normality with the Shapiro-Wilk, D'Agostino-Pearson, or Kolmogorov-Smirnov tests. The statistical significance of differences between/among groups was assessed by paired or unpaired two-tailed Student's *t*-tests, or ANOVA, as indicated. Time-series experiments were analyzed using mixed-design ANOVA or linear mixed-effects models when data were missing. For data with a non-normal distribution, the non-parametric Wilcoxon rank sum test or the Kruskal-Wallis test was used. For comparisons of group proportions, the chi-square test or Fisher's exact test (for samples with expected frequencies below 5) was used. *p*-values < 0.05 were considered significant. For the analysis of relationships between variables, simple linear regression and Pearson correlation were used. Data are presented as means ± standard error of the mean (SEM). All statistical tests and results are reported in Supplementary Table 1.

## 3 Results

### 3.1 Characterization of the *in vitro* hiPSC-derived neuromuscular co-culture system

To assess whether mHTT transmission can contribute to skeletal muscle pathology in patients with HD, we designed an *in vitro*, isogenic hiPSC-derived neuromuscular co-culture system. The hiPSC line (hDFa90/1.2) used in this study was generated from the human dermal fibroblast line C-013-5C (Thermo Fisher). Pluripotency was confirmed by positive staining for the pluripotency markers Nanog, SSEA-4, OCT4, and TRA-1-81 using immunocytochemistry (Supplementary Figure 1A). The karyotype of the cells was evaluated by whole-genome SNP analysis and all lines showed a normal karyotype (Supplementary Figure 1B). Using the hPSC Scorecard assay, we showed that the hiPSCs could differentiate into cells of all three germ layers (Supplementary Figure 1C). From the hDFa90/1.2 line, we generated two transgenic cell lines, one bearing a DOX-inducible *NGN2* transgene (Russell et al., 2018), which encodes a pro-neuronal transcription factor, and one bearing a DOX-inducible *MyoD* transgene, coding for a pro-skeletal muscle transcription factor (Rose et al., 2022). We differentiated the *NGN2* and *MyoD* iPSCs using a motor neuron- and a myotube-specific differentiation protocol, respectively (Supplementary Figure 2A). To confirm that the *NGN2* and *MyoD* iPSCs did indeed differentiate into motor neuron- and myotube-like cell types, we assessed the expression of motor neuron- and myotube-specific mRNA markers (NKX6.1, ISL1, and ChAT for motor neurons and MyoG, MUSK, and TPM2 for myotubes. We found that motor neuron-specific mRNAs were selectively expressed in NGN2-expressing cells while myotube-specific mRNAs were specifically expressed in MyoD-expressing cells (Supplementary Figure 2B). Next, we established neuromuscular co-cultures using a two-step differentiation protocol (Supplementary Figure 2A). Using immunofluorescence staining, we found that on DCC 7, NGN2-expressing neurons were positive for the pan-neuronal marker MAP2 and the motor neuronal marker ISL1 (Supplementary Figure 2C), and most NGN2-expressing neurons were also positive for ISL1 (Supplementary Figure 2D). In contrast, the expression of these proteins was not detected in myotubes; instead, MyoD-positive myotubes expressed the myotube-specific marker MHC1, which was absent from NGN2-positive neurons (Supplementary Figure 2D). Because motor neurons specifically express the neurotransmitter ChAT, we also stained the co-cultures for this marker at DIV 21 and found that it was selectively expressed in most of the NGN2-positive neurons (Supplementary Figures 2E, F). Taken together, these findings showed that NGN2-expressing hiPSCs differentiated into motor neuron-like cells, whereas MyoD-expressing hiPSCs differentiated into myotube-like cells.

### 3.2 A novel neuromuscular co-culture system to study mHTT transmission across functional NMJs

We have previously shown that mHTT transmission occurs over time and likely depends on the presence of synaptic connections (Pecho-Vrieseling et al., 2014). To visualize mHTT transmission from motor neurons to myotubes over weeks in the same co-culture, we designed a system that was compatible with high-throughput, low-resolution, live-cell imaging. To this end, we used the neuronal *NGN2* hiPSC line to generate a double-transgenic cell line expressing exon1 of the *HTT* gene harboring 72 CAG triplets (pathogenic) fused to a Cre sequence lacking a nuclear localization signal (NGN2;HTTEx1Q72-Cre). Additionally, we generated an isogenic MyoD-expressing myotube cell line bearing a LoxP-GFP construct (MyoD;LoxP-GFP), which would allow the visualization of the uptake of mHTTEx1-Cre released from the neurons (Supplementary Figure 3A). We assessed HTTEx1Q72-Cre transgene expression via western blotting using an anti-HTT exon 1 antibody (Supplementary Figure 3B). To determine whether the expressed HTTEx1Q72-Cre protein formed aggregates, we differentiated the hiPSC lines into neurons. On day 1 of differentiation, we observed that there was a loss of pluripotency, as evidenced by a decrease in OCT4 expression (Supplemental Figure 3C). After 7 and 21 days of differentiation, we assessed HTTEx1Q72-Cre aggregation using the EM48 antibody, which has a high affinity for the aggregated form of HTT. We measured EM48 fluorescence intensity as a read-out for aggregation. No aggregation was observed at DIV 7 and 21 (Supplementary Figure 3D).

To test the Cre-Lox system, we electroporated MyoD;LoxP-GFP hiPSCs with the HTTEx1-Cre construct and the NGN2;HTTEx1Q72-Cre hiPCSs with a LoxP-mCherry plasmid, resulting in GFP- and mCherry-expressing cells, respectively. In the absence of Cre, GFP expression was never detected in MyoD;LoxP-GFP hiPSCs (*n* = 3; Supplementary Figure 3E).

These results demonstrated that the Cre-Lox system was functional and could thus be used to investigate the motor neuron-to-myotube transmission of mHTTEx1Q72.

**Figure 1:**
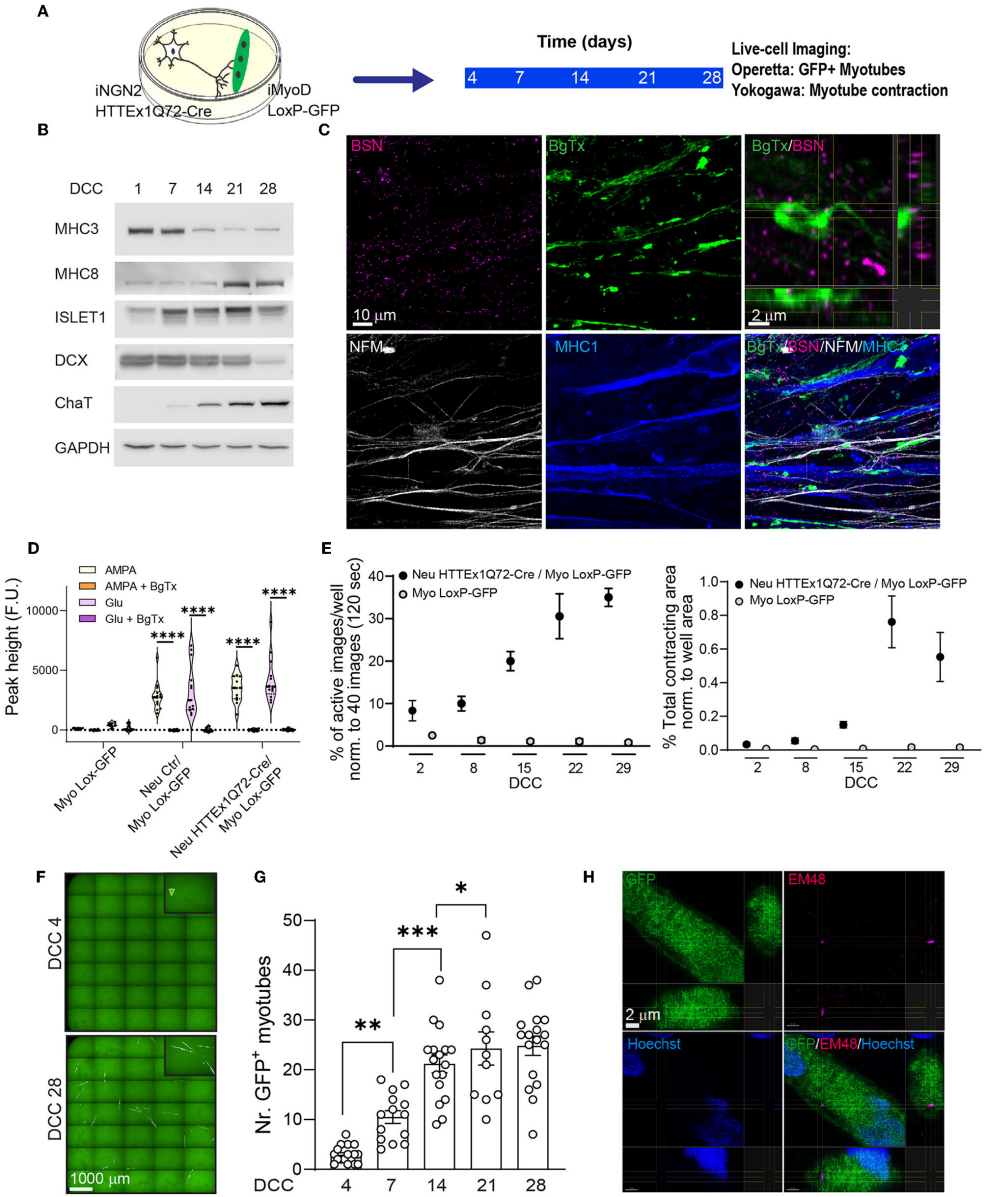
The transmission of HTTEx1Q72 from neurons to muscle cells in hiPSC-derived neuromuscular co-cultures. **(A)** The experimental approach used to follow in parallel the development of functional NMJ activity and the transmission of HTTEx1Q72-Cre from neurons to myotubes bearing a LoxP-GFP sequence employing live-cell, high-throughput imaging. **(B)** Representative western blot of developmental markers for myotubes (MHC3 = embryonic myosin, MHC8 = postnatal myosin) and motor neurons (DCX, ISL1, and ChAT) with increasing DCC. **(C)** IF images of neuromuscular synapses in Neu HTTEx1Q72-Cre/Myo LoxP-GFP co-cultures on DCC 21. Top right: orthogonal view of the presynaptic active zone marker BSN in close apposition to the postsynaptic marker BgTx (labels AChRs on myotubes). NFM labels axons, and MHC1 is a pan-myosin marker. **(D)** Graph depicting the intracellular calcium peaks recorded in myotubes with exposure of the cultures to the neuron-specific glutamate receptor agonists AMPA and glutamate and in the presence of the acetylcholine receptor blocker BgTx (Myo Lox-GFP = monoculture; Neu Ctr/Myo Lox-GFP and Neu HTTEx1Q72-Cre/Myo Lox-GFP are co-cultures). **(E)** The percentage of images showing myotube contractions (left graph) and the total myotube contracting area (right graph) obtained from a single well of a 96-well plate of neuromuscular co-culture and a muscle-only monoculture with increasing DCC [*n* = 9 wells/time point in three independent cultures (on DCC 2, *n* = 6 from 2 cultures)]. Two-way mixed-design ANOVA (without DCC 2): time-dependent significant differences between co-cultures and muscle-only monocultures (*p* = 1.21e-10 left panel, *p* = 0.0001 right panel). *Post-hoc* one-way repeated measures ANOVA: significant increase with time in both parameters for co-cultures (*p* = 0.0003 left panel, *p* = 0.0001 right panel) and no significant effect for muscle-only cultures. **(F)** Live-cell fluorescence image obtained with the Operetta high-throughput imaging system showing GFP+ myotubes on DCC 4 (arrowhead) and DCC 28. **(G)** The number of GFP+ myotubes obtained using Operetta in Neu HTTEx1Q72-Cre/Myo LoxP-GFP co-culture with increasing DCC (*n* = 11–17 wells/time point from at least four independent experiments); ***p = 0.0008, **p = 0.002, *p = 0.015 (linear mixed model, Tukey's correction). **(H)** Orthogonal view of an IF image showing a mEM48+ HTT aggregate in the cytoplasm of a GFP+ myotube. Nuclei are labeled with Hoechst (blue). AChRs, acetylcholine receptors; BgTx, α-bungarotoxin; BSN, bassoon; ChAT, choline acetyltransferase; DC, doublecortin; DCC, days of co-culture; IF, immunofluorescence; MHC, myosin heavy chain; NFM, neurofilament M; NMJ, neuromuscular junction. All averaged data are shown as means ± SEM. ****p ≤ 0.0001.

Next, we established neuromuscular co-cultures with the above cell lines (Figure 1A). The molecular maturation of the myotubes and neurons in co-culture was assessed by western blotting on DCC 1, 7, 14, 21, and 28 using myotube-specific antibodies (MHC3 and MHC8) as well as neuronal-specific (DCX, a neuronal precursor marker) and motor neuron-specific (ISL1 and ChAT) antibodies. We found that, with increasing co-culture time, there was a decrease in the levels of precursor markers and an increase in those of postnatal markers (Figure 1B). The two cell types are hereafter referred to as Neu HTTEx1Q72-Cre for the neurons and Myo LoxP-GFP for the myotubes.

To investigate the formation of synaptic connections between neurons and myotubes, we employed immunofluorescence staining to identify neuromuscular junctions (NMJs), which represent the synaptic connections between motor neuron axons and muscles. Motor neuron axons form the presynaptic structure and muscles the postsynaptic structure of the synapse. The results revealed that NMJs formed between Neu HTTEx1Q72-Cre and Myo LoxP-GFP-expressing cells. There was a close apposition between the neuronal presynaptic active zone marker BSN and the postsynaptic marker α-BgTx, which labels AChR on myotubes (Figure 1C). Muscle cell contraction relies on a rise in cytosolic calcium concentrations, a process that is dependent on the NMJ. Following a neuronal stimulus, acetylcholine (ACh) is released from presynaptic motor neuron terminals and activates AChRs on the myotubes, resulting in their depolarization, and, consequently, the opening of Ca^2+^ channels; this, in turn, leads to an increase in cytosolic Ca^2+^ in myotubes, which activates the muscle contractile apparatus (Kuo and Ehrlich, 2015). Here, to assess whether this mechanism was active in our cultures, we treated the co-cultures on DCC 21 with AMPA or glutamate, well-characterized neuronal glutamate receptor agonists that depolarize neurons and trigger Ach release. This treatment resulted in elevated intracellular Ca^2+^ levels in myotubes. Importantly, this increase in cytosolic Ca^2+^ was blocked following the addition of the AChR antagonist BgTx to the medium (Figure 1D). The above experiments demonstrated the presence of functional NMJs in Neu HTTEx72-Cre/Myo LoxP-GFP co-cultures.

To gain better insight into the progressive development of these NMJs, we followed myotube contractions for 29 days in the same wells of either co-cultures or Myo LoxP-GFP monocultures using live-cell imaging. The results revealed that there was a temporal increase in both myotube activity and contracting area in co-cultures but not monocultures (Figure 1E). As seen with cytosolic Ca^2+^ concentrations, these contractions were also blocked when BgTx was added to the co-cultures, thus revealing their dependence on NMJ activity (Supplementary Figure 4). Together, these data validated the suitability of this system for addressing whether HTTEx1Q72-Cre can be transmitted from motor neurons to myotubes across functional NMJs.

### 3.3 HTTEx1Q72-Cre was transmitted from motor neurons to myotubes, and this was linked to neuromuscular maturation

To gauge HTTEx1Q72-Cre transmission, we performed high-throughput, live-cell fluorescence imaging in the same wells from DCC 4 to DCC 28. The first GFP+ myotubes appeared on DCC 4 and their number increased with increasing co-culture duration until DCC 21, after which the numbers remained stable (Figures 1F, G). This timing correlated with the establishment of functional NMJs (Figure 1E). Based on mEM48 staining on DCC 7 and DCC 21, the aggregated form of HTT was not detected in HTTEx1Q72-Cre-positive motor neurons (Supplementary Figure 3D). When we stained the co-cultures on DCC 28, we detected the presence of EM48-positive aggregates in only a few GFP+ myotubes (Figure 1H; Supplementary Figure 5). This finding suggested that mHTT transmission likely occurs in a non-aggregated form, followed by aggregation in myotubes.

To verify that HTTEx1Q72-Cre neuromuscular transmission requires direct cell–cell contact and does not occur via the culture medium, we placed a bottomless two-chamber cell culture insert in one dish to allow the physical separation of HTTEx1Q72-Cre motor neurons and LoxP-GFP myotubes, while the medium was shared between the two cell types. After cell attachment, the inserts were removed (DCC 1). The surface between the insert was not coated to prevent the movement of the cells as well as the extension of the axons to the myotubes. GFP+ myotubes were never observed in these co-cultures (Supplementary Figures 6A, B).

Taken together, using this novel neuromuscular co-culture system, we have shown that mHTT is transferred from motor neurons to myotubes in a progressive manner, that the transferred protein species are likely to be in a non-aggregated form, and that this transfer depends on neuromuscular connectivity and possibly also NMJ activity.

### 3.4 Neuromuscular co-cultures in MFDs revealed HTTEx1Q72 transmission across NMJs

**Figure 2:**
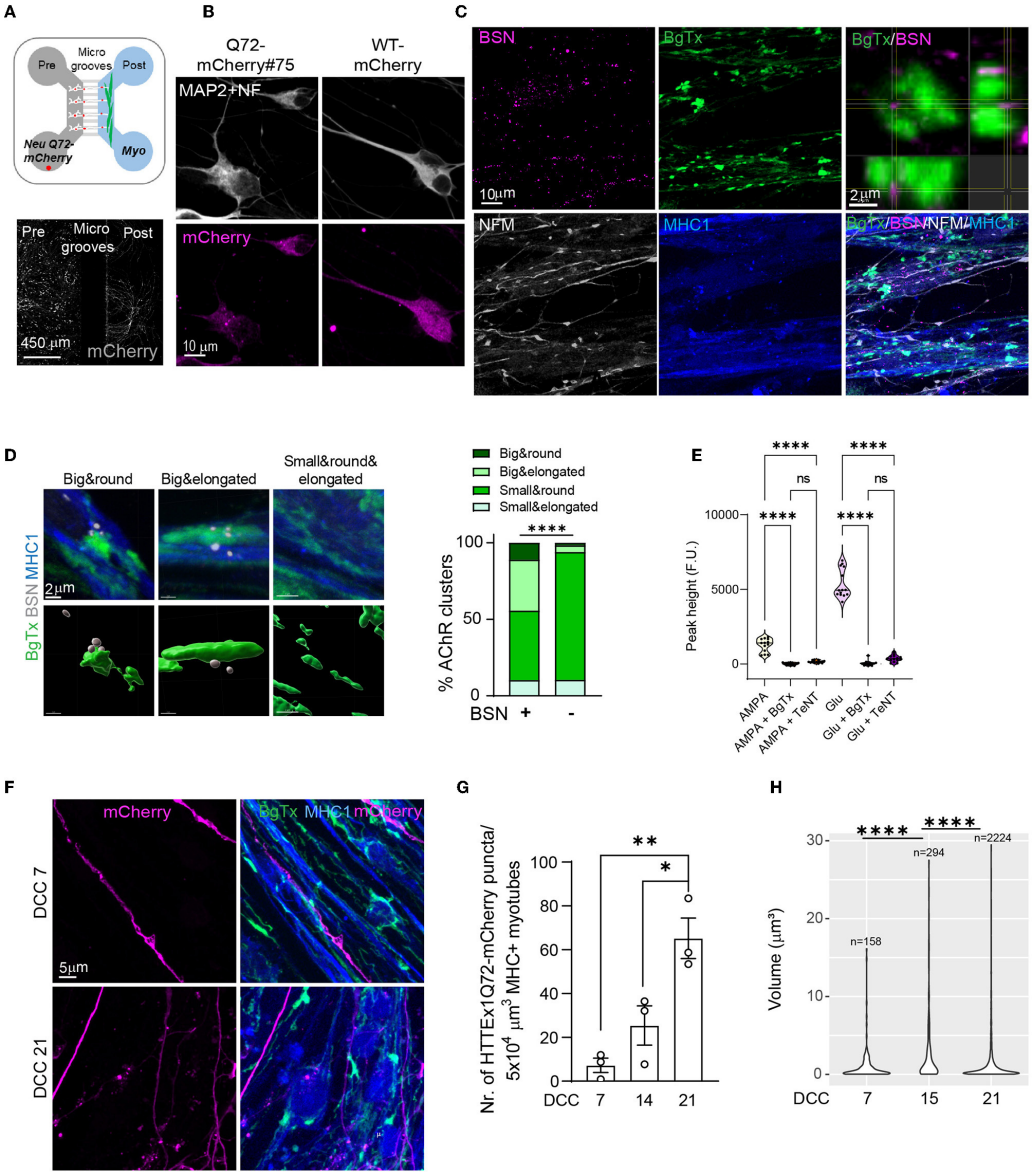
The transmission of HTTEx1Q72 across the NMJ in Neu HTTExQ72-mCherry clone#75/myotube co-cultures in MFDs (**A**, upper panel). Schematic of the MFD co-culture setting: Neu HTTEx1Q72-mCherry cells (white with red dots) in the presynaptic chamber and myotubes (green) in the postsynaptic chamber. Neurons extend their axons through the microgrooves to the myotubes. (**A**, lower panel) IF staining for mCherry (white) in a MFD shows Neu HTTEx1Q72-mCherry cells in the presynaptic chamber and the extension of their axons to the postsynaptic compartment. **(B)** IF images of neurons expressing HTTEx1Q72-mCherry (punctate mCherry staining) and WT-mCherry (diffuse staining) on DCC 7. **(C)** IF images of neuromuscular synapses in Neu HTTEx1Q72-mCherry clone#75/Myo LoxP-GFP co-cultures in an MFD on DCC 21. Top right: an orthogonal view of the close apposition between BSN and BgTx. (**D**, left) IF images of the four types of AChR clusters identified on neuron-myotube co-cultures (upper panels) and the corresponding surfaces created in Imaris: AchR clusters are labeled with BgTx (green), and BSN is shown in white (lower panels). (**D**, right) Percentage distribution of the AChR cluster types found on myotubes when associated with (+) or not (–) with BSN [*n* = 1040 for (+); *n* = 11745 for (–)]; ****p < 2.2 × 10^−16^ (χ^2^ test). **(E)** Graph depicting the intracellular calcium peaks recorded in myotubes upon exposure of the cultures to the neuron-specific glutamate receptor agonists AMPA or glutamate and in the presence of the acetylcholine blocker BgTx or the neurotransmitter release blocker TeNT. **(F)** IF staining in the myotube compartment shows the presence of HTTEx1Q72-mCherry+ axons on DCC 7 and axons and puncta on DCC 21 in MHC1+ myotubes. **(G)** The number of HTTEx1Q72-mCherry puncta in myotubes from DCC 7 to DCC 21 (*n* = 3 independent co-cultures/time point; data point: mean of 15 images); *p = 0.02, **p = 0.004 (one-way ANOVA, Tukey's correction for multiple comparisons). **(H)** Violin plot of the volume of individual HTTEx1Q72-mCherry puncta localized in myotubes from DCC 7 to DCC 21. *n* is the number of puncta found in myotubes across all images at a given time point. ****p ≤ 1 × 10^−9^ (Kruskal-Wallis test, Benjamini-Hochberg correction). AChR, acetylcholine receptor; BgTx, α-bungarotoxin (labels AchRs); BSN, bassoon; DCC, days of co-culture; IF, immunofluorescence; MHC, myosin heavy chain; MFD, microfluidic device; NFM, neurofilament M; NMJ, neuromuscular junction; TeNT, tetanus neurotoxin. All averaged data are shown as means ± SEM.

To confirm that HTTEx1Q72 is transmitted across the NMJ, we established neuromuscular co-cultures in MFDs. In these devices, motor neurons and myotubes are co-cultured in fluidic isolated compartments that are connected via microgrooves through which only motor neuron axons can grow. This assures that the only connections that can be formed between motor neurons and myotubes are NMJs (Figure 2A). Using this system, we followed HTTEx1Q72 transmission using mCherry instead of Cre as a tag (Figure 2A, lower panel). The mCherry tag allows the visualization of the HTTEx1Q72 peptide, thereby gaining insight into its behavior, relating to, for example, its subcellular localization and aggregation. We generated a second isogenic NGN*2*-expressing line harboring HTTEx1Q72 fused to mCherry (Neu HTTEx1Q72-mCherry; Supplementary Figure 3A). We assessed transgene expression by western blot using an anti-HTT exon 1 antibody and identified iPSC clones with different levels of HTTEx1Q72-mCherry expression (Supplementary Figure 3B). After 7 and 21 days of differentiation, we evaluated mHTT aggregation using the EM48 antibody and found that mHTT aggregation increased with increasing expression of the HTTEx1Q72 protein in Neu HTTEx1Q72-mCherry clones (Supplementary Figure 3D).

For the first experiment, we used the Neu HTTEx1Q72-mCherry high-expressing cell line derived from clone#75. This line showed punctate mCherry staining (Figure 2B, left panel) and the puncta co-localized with the EM48 antibody (Supplementary Figure 7). Clone#75 neurons were co-cultured with Myo LoxP-GFP cells [we continued using this myotube line as GFP was not expressed in the absence of Cre and thus did not interfere with the analysis (Supplementary Figure 3E)]. As a control, we generated an isogenic *NGN2* hiPSC line expressing only mCherry (Neu mCherry), which showed a diffuse mCherry expression pattern (Supplementary Figure 3A; Figure 2B, right panel).

The mCherry labeling of Neu HTTEx1Q72-mCherry cells grown in MFDs showed that they projected their axons from the presynaptic neuronal compartment to the postsynaptic myotube compartment (Figure 2A, lower panel). NMJs were found to be established in the myotube compartment, as visualized by IF staining of BSN and AChR appositions on DCC 21 (Figure 2C). AChR clusters on the surface of myotubes can be classified based on their shapes (Lutz et al., 2020). We performed a detailed shape analysis of these clusters on DCC 21 and classified them into four categories—small and elongated, small and round, big and elongated, and big and round (Supplementary Figures 8A–C). When we compared the clusters in myotubes in mono- vs. co-cultures, we found that the clusters of the small-and-round type were the most abundant in both cultures; however, the density of big-type clusters (both elongated and round) was significantly higher in the co-cultures than in the monocultures (Supplementary Figures 8D, E). The big-cluster types are thus likely those constituting the NMJs. Supporting this notion, we found that the big-cluster types were overrepresented among the clusters associated with the presynaptic marker BSN compared with that in clusters free of BSN staining (Figure 2D). We also stimulated the motor neurons in these cultures with AMPA and glutamate and found that cytosolic Ca^2+^ levels were increased in the myotubes (Figure 2E); This increase was blocked when BgTx was added to the myotube compartment or when TeNT, well-known as a blocker of neurotransmitter release, was added to the neuronal compartment (Figure 2E). These observations indicated that functional NMJs form between Neu HTTEx1Q72-mCherry cells and MyoD-expressing myotubes in MFDs.

Next, using immunofluorescence labeling, we found that the HTTEx1Q72-mCherry protein was present in myotubes in the postsynaptic compartment, indicating transmission from neurons (Figure 2F). Like with HTTEx1Q72-Cre, we observed that HTTEx1Q72-mCherry transmission to myotubes increased continuously from DCC 7 to DCC 21, as determined via the quantification of the number of HTTEx1Q72-mCherry puncta in the myotubes (Figure 2G). We analyzed the volume of HTTEx1Q72-mCherry puncta over time and identified a dynamic change in size distribution across the sampling time points (Figure 2H). On DCC 14, larger aggregates with sizes above 15 μm^3^ appeared, while there was a big decrease in the contribution of smaller assemblies compared with that on DCC 7. On DCC 21, small assemblies with sizes below 1 μm^3^ again made a major contribution, together with a persistent presence of bigger aggregates. This suggested that new molecules arrive in the muscle as small assemblies and that aggregation subsequently occurs over time. Together, these findings provide evidence that HTTEx1Q72 is transmitted across the NMJ, most likely in the form of small protein complexes.

### 3.5 The load of HTTEx1Q72-mCherry was correlated with increasing neuromuscular connections

**Figure 3:**
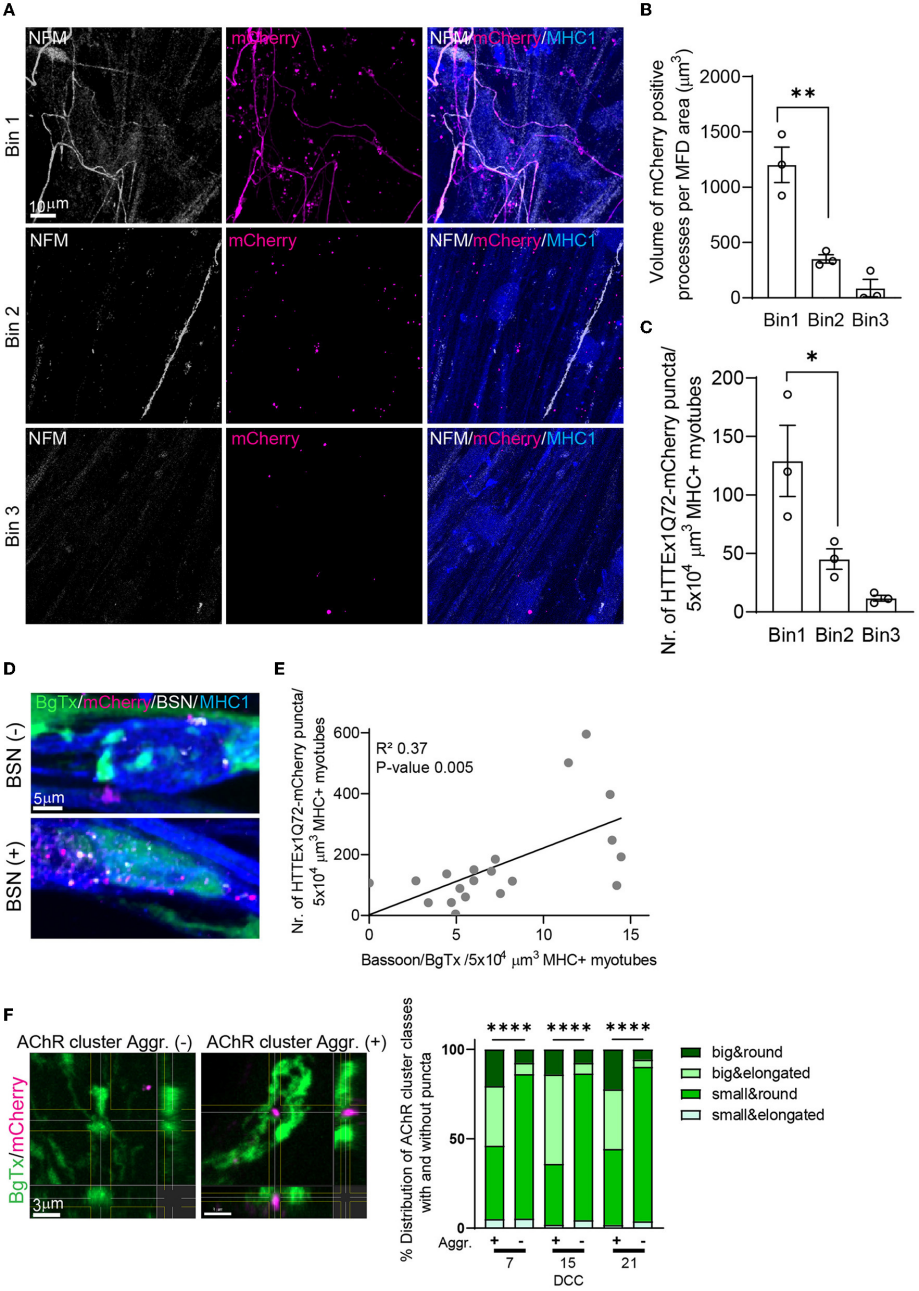
Increasing the NMJ density enhances the neuromuscular transmission of HTTExQ72. **(A)** IF image of NFM+ axons and HTTEx1Q72-mCherry in bin 1, bin 2, and bin 3 of the myotube compartment in co-cultures with Neu HTTEx1Q72-mCherry clone#75 on DCC 21. **(B)** The total volume of mCherry-positive neuronal processes that crossed to the myotube compartment normalized to the MFD area in each bin (*n* = 3 independent co-cultures; data point: mean of five images); **p = 0.003. **(C)** The number of HTTEx1Q72-mCherry aggregates inside myotubes in each bin (*n* = 3 independent co-cultures; data point: mean of five images); *p = 0.04 (one-way ANOVA, Tukey's correction). **(D)** IF staining of Neu HTTEx1Q72-mCherry/myotube co-cultures showing mHTTEx1 in regions with a low **(top)** or high **(bottom)** number of BSN–BgTx appositions. **(E)** Correlation between the number of NMJs (defined as BSN–BgTx appositions on MHC1+ myotubes) and the number of HTTEx1Q72-mCherry puncta (*n* = 20 images from 3 MFDs, simple linear regression). (**F**, left panels) IF staining of AChR clusters in the absence of (–) (>0.05 μm) or in close proximity to (+) (within 0.05 μm) HTTEx1Q72-mCherry puncta. (**F**, right panel) The distribution of AChR cluster types when associated (+) (within 0.05 μm) or not (–) with HTTEx1Q72-mCherry from DCC 7 to DCC 21 [DDC 7: *n* = 39 (+), *n* = 10,183 (–); DCC 15: *n* = 50 (+), *n* = 5,161 (–); DCC 21: *n* = 277 (+), *n* = 10,263 (–)]; ****p < 0.0001 (Fisher's exact test for DCC 7 and DCC 15, χ^2^ test for DCC 21). AChR, acetylcholine receptor; BgTx, α-bungarotoxin (labels AChRs); BSN, bassoon; DCC, days of co-culture; IF, immunofluorescence; MFD, microfluidic device; MHC, myosin heavy chain; NFM, neurofilament M; NMJ, neuromuscular junction. All averaged data are shown as means ± SEM.

One of the presymptomatic pathologies in patients with HD is the loss of functional neuronal connectivity, which first arises in the cortico-striatal pathway and then progresses to cortical and other subcortical brain regions (Tabrizi et al., 2011, 2013; Poudel et al., 2014, 2019; Harrington et al., 2015). Interestingly, the most vulnerable brain regions form a selective network that has higher connectivity than other brain regions (van den Heuvel and Sporns, 2011), a so-called “rich club”. To assess whether the number of HTTEx1Q72-mCherry puncta in myotubes increased with increasing NMJ density, we divided the postsynaptic compartment of MFDs into three bins, with bin 1 closest to and bin 3 farthest from the microgrooves. The size of the area containing neuronal processes was greatest in bin 1 and lowest in bin 3 (Figures 3A, B; Supplementary Figure 9A). Similarly, we found that the number of HTTEx1Q72-mCherry puncta in myotubes on DCC 21 was highest in bin 1 and steeply decreased toward bin 3 (Figures 3A, C). Interestingly, when we compared the distribution in the bins at the different time points, we observed a slower increase in the number of puncta in bin 2 compared with that in bin 1 (Supplementary Figure 9B). This delay in HTTEx1Q72-mCherry accumulation in myotubes can be explained by the fact that axons will arrive slightly later in bin 2 than in bin 1, followed by delayed NMJ formation. Further validating our assumption that HTTEx1Q72-mCherry proteins reach the myotubes via NMJs, we found a positive correlation between the density of NMJs (BSN-BgTx complexes) and the number of HTTEx1Q72-mCherry puncta in myotubes (Figures 3D, E).

We have previously shown that mHTT is transmitted from mouse cells in HD-derived mouse organotypic brain slices (OTBS) to human stem cell-derived neurons (h-neurons). During transmission, mHTT was seen to co-localize with the presynaptic marker synaptophysin and post-synaptic density protein-95 (PSD-95) in neurons (Pecho-Vrieseling et al., 2014). In our neuromuscular co-cultures, we also observed that ~20% of HTTEx1Q72-mCherry puncta were associated with postsynaptic AChRs (Supplementary Figure 9C). Interestingly, approximately 60% of the AChR clusters associated with HTTEx1Q72-mCherry puncta were of the big type, while among the AChR clusters with no HTTEx1Q72-mCherry expression, only ~10% were of the big type (Figure 3F). The big-type clusters likely represent clusters incorporated in the NMJs given that the number of these cluster types was increased in the presence of neurons and in association with the presynaptic marker BSN (Supplementary Figure 8E; Figure 2D). In summary, we found that there was a positive correlation between neuromuscular connectivity and the HTTEx1Q72-mCherry load in myotubes and identified a preferential association between HTTEx1Q72-mCherry and AChR clusters in NMJs.

### 3.6 Blocking neurotransmitter release decreased the neuromuscular transmission of mHTTEx1

**Figure 4:**
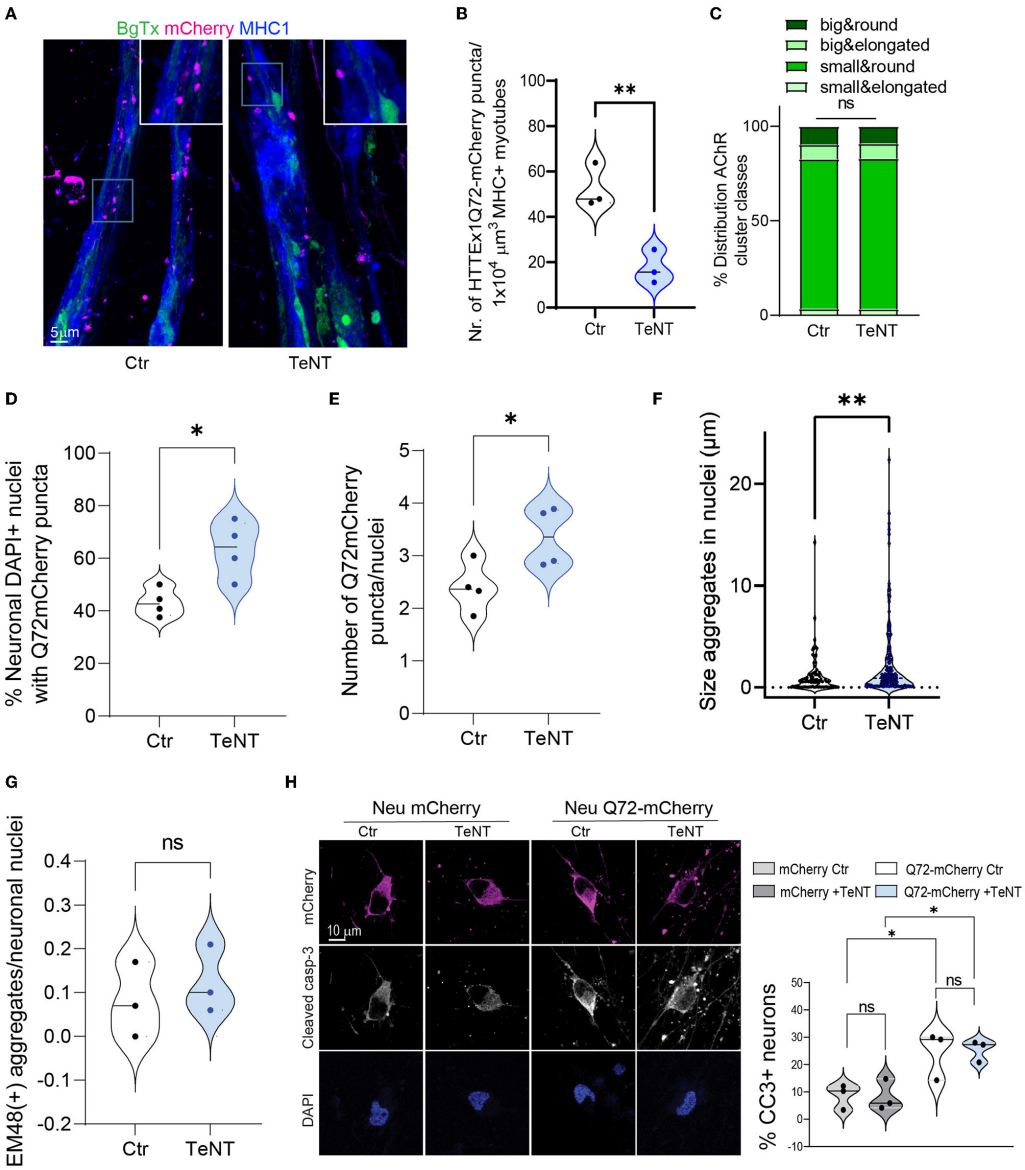
Blocking synaptic activity reduces the transmission of HTTEx1Q72 from neurons to myotubes. **(A)** IF staining of Neu HTTEx1Q72-mCherry clone#75/myotube co-cultures on DCC 21. The co-cultures were exposed or not (control) to 2 μg/ml of TeNT from DCC 10. **(B)** The number of HTTEx1Q72-mCherry puncta in MHC1+ myotubes in control and TeNT-treated co-cultures (*n* = 3, one data point corresponds to one independent co-culture and is the mean of five images; **p = 0.0076, Student's *t*-test). **(C)** The distribution of AChR cluster types found on myotubes in control and TeNT-treated co-cultures (*n* = 1,304 for control, *n* = 882 for TeNT; ns, χ^2^ test). **(D)** The percentage of neuronal nuclei bearing HTTEx1Q72-mCherry puncta in control or TeNT-treated co-cultures on DCC 21 (each dot represents the average of 10 pictures from four independent experiments; *p = 0.0156, Student's *t*-test). **(E)** The number of HTTEx1Q72-mCherry puncta per neuronal nucleus in control or TeNT-treated co-cultures on DCC 21 (each dot represents the average of 10 pictures from four independent experiments; *p = 0.04, Student's *t*-test). **(F)** The area of HTTEx1Q72-mCherry puncta in neuronal nuclei in control and TeNT-treated co-cultures (*n* = 37 for control, *n* = 141 for TeNT; *p = 0.0061, Student's *t*-test). **(G)** The number of mEM48+ aggregates in neuronal nuclei from control or TeNT-treated co-cultures on DCC 21 (each dot represents the average of 20 neurons from three independent experiments; *p* = 0.5511, Student's *t*-test). **(H)** IF staining for cleaved caspase-3 in Neu mCherry and Neu HTTEx1Q72-mCherry neurons in control or TeNT-treated cultures. Shown are the percentages of cleaved caspase-3-expressing mCherry+ neurons under all conditions [each dot represents the average of 91 untreated Neu mCherry neurons (mCherry Ctr); 102 Neu mCherry neurons treated with TeNT (mCherry +TeNT); 48 untreated HTTEx1Q72-mCherry neurons (Q72-mCherry Ctr); and 60 HTTEx1Q72-mCherry neurons treated with TeNT (Q72-mCherry+TeNT)]; all results are from three independent experiments: mCherry Ctr vs. mCherry +TeNT, *p* = 0.9998; Q72-mCherry Ctr vs. Q72-mCherry +TeNT, *p* = 0.9978; mCherry Ctr vs. Q72-mCherry Ctr, *p* = 0.0501; and mCherry +TeNT vs. Q72mCherry +TeNT, *p* = 0.0351 (one-way ANOVA). All averaged data are shown as means ± SEM. AChR, acetylcholine receptor; DCC, days of co-culture; IF, immunofluorescence; MHC, myosin heavy chain; TeNT, tetanus neurotoxin.

It has been shown both in OTBS/h-neuron co-cultures and *in vivo* in *Drosophila* that the neuron-to-neuron transmission of mHTT is markedly inhibited when the SNARE-dependent fusion of synaptic vesicles with the presynaptic membrane and the subsequent release of their contents are blocked (Pecho-Vrieseling et al., 2014; Babcock and Ganetzky, 2015). Consequently, we applied TeNT, which cleaves SNARE proteins (Dong et al., 2019), to Neu HTTEx1Q72-mCherry clone#75/myotube co-cultures on DCC 10, and found that the AMPA-dependent increase in intracellular Ca^2+^ levels in myotubes was blocked (Figure 2E). This confirmed that TeNT effectively blocked NMJ activity. Like with neuron-to-neuron transmission, we observed that the neuromuscular transmission of HTTEx1Q72-mCherry was significantly decreased, as evidenced by the decrease in the number of mCherry puncta within the myotubes on DCC 21 with TeNT treatment (Figures 4A, B). Interestingly, the proportions of AChR cluster types were similar between the treatment and control conditions, suggesting that NMJ structure was not affected (Figure 4C). This indicated that the observed effect was due to the blockage of presynaptic neuronal terminals and not the reorganization of postsynaptic structures.

We hypothesized that the synaptic transmission of mHTTEx1 can be regarded as a clearance mechanism by which cells discard toxic protein species. Accordingly, we addressed whether blocking this rescue process using TeNT treatment would increase the number of mHTTEx1-mCherry puncta in neurons. Previously, we have shown that mHTTEx1 transmitted from mouse cells to human stem cell-derived neurons first appeared as cytoplasmic aggregates followed, with time, by the emergence of aggregates in the nucleus. Additionally, nuclear aggregation was correlated with the timing of the occurrence of pathological changes in the human neurons (Pecho-Vrieseling et al., 2014). In our co-culture system, TeNT treatment resulted in an increase in the number of DAPI+ nuclei containing HTTEx1Q72-mCherry puncta, as well as an increase in the number and size of neuronal intra-nuclear assemblies (Figures 4D–F). No difference was detected in the number of HTTEx1Q72 puncta positive for EM48 between control and TeNT-treated cultures (Figure 4G). EM48 only recognizes the aggregated form of mHTT and thus the mCherry puncta that we observed might either contain a non-aggregated form of mHTT or an aggregated form with a conformation that is not recognized by EM48 (Bayram-Weston et al., 2016). We also performed caspase staining to assess cell death in the cultures. TeNT treatment did not cause an increase in cell death compared with that in the control condition. In contrast, cell death was significantly increased in HTTEx1Q72-mCherry-expressing cells relative to that in control cultures; however, even in these cultures, cell death was not aggravated by TeNT treatment (Figure 4H). Taken together, these results indicated that blocking neurotransmitter release with TeNT results in reduced NMJ activity, as indicated by a reduction in calcium concentrations in myotubes. In these co-cultures, we also observed a reduction in neuromuscular transmission of HTTEx1Q72-mCherry particles. Meanwhile, blocking neurotransmitter release from neurons results in an increase in the number and size of HTTEx1Q72 intranuclear puncta in neurons, but this is not accompanied by an increase in aggregation or cell death within the time window assessed. A culture duration of 21 days might have been insufficient for these pathologies to develop.

### 3.7 Increased neuronal activity enhanced the neuromuscular transmission of mHTTEx1

**Figure 5:**
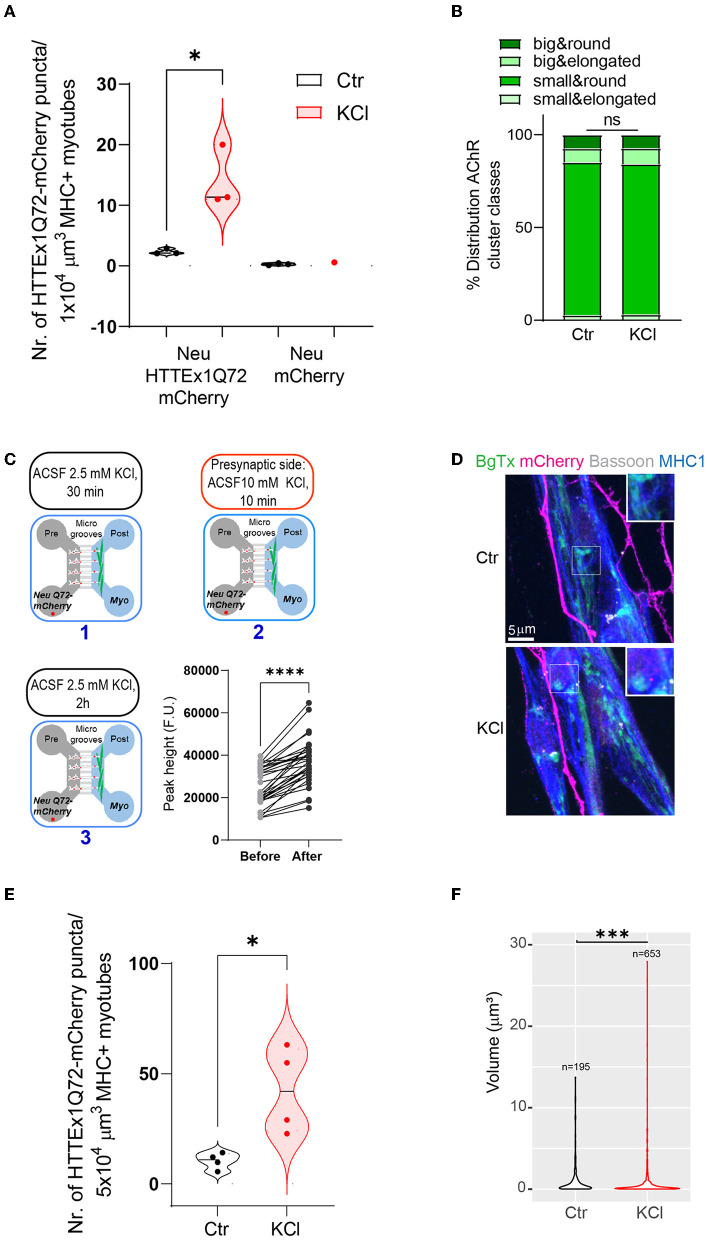
Enhancing synaptic activity increases the transmission of HTTEx1Q72 from neurons to myotubes. **(A)** The number of mCherry aggregates in MHC1+ myotubes after co-culture with either Neu HTTEx1Q72-mCherry cells or Neu mCherry cells and exposure to 2.5 mM KCl (Ctr) or 10 mM KCl (*n* = 3; one data point corresponds to one independent culture and is a mean of five images; *p = 0.016, Student's *t*-test). **(B)** The distribution of AChR cluster types found on myotubes under control conditions or following treatment with 10 mM KCl (*n* = 1,091 for control, *n* = 1,377 for 10 mM KCl; ns, χ^2^ test). (**C**, upper panels and lower left panel) A schematic outline of the experimental approach using MFDs. ACSF with 2.5 mM KCl (1) was added to both the neuronal and myotube compartments for 30 min. ACSF with 10 mM KCl (2) was added only to the neuronal compartment for 10 min. ACSF with 10 mM KCl was changed to ACSF with 2.5 mM KCl (3). The cultures were fixed after 2 h (**C**, lower right panel) Graph depicting intracellular calcium levels recorded in myotubes cultured in MFDs following exposure of only neurons to 2.5 mM KCl and 10 mM KCl (*n* = 33 cells, ****p = 0.0001, Student's paired *t*-test). **(D)** IF images depicting HTTEx1Q72-mCherry puncta in MHC1+ myotubes in cultures exposed to 2.5 mM KCl (Ctrl) or 10 mM KCl (KCl). The inserts correspond to a higher magnification of a representative region. **(E)** The number of HTTEx1Q72-mCherry puncta in MHC1+ myotubes in control (treated with 2.5 mM KCl) co-cultures or 10 mM KCl-treated co-cultures (*n* = 4; one data point corresponds to one independent co-culture and is a mean of 10 images; *p = 0.018, Student's *t*-test). **(F)** Violin plot of the volume of HTTEx1Q72-mCherry puncta in control and 10 mM KCl-treated co-cultures (*n* indicates the total number of puncta analyzed; ***p = 0.0008, Wilcoxon rank sum test). All averaged data are shown as means ± SEM. AChR, acetylcholine receptor; ACSF, artificial cerebral spinal fluid; MFD, microfluidic device; MHC, myosin heavy chain.

As inhibition of synaptic vesicle release reduced HTTEx1Q72-mCherry transmission, we asked if the opposite effect can be obtained via the depolarization of neurons, which triggers increased action potential firing and synaptic vesicle release (Rienecker et al., 2020). We depolarized neurons by exposing Neu HTTEx1Q72-mCherry clone#72/myotube co-cultures on DCC 21 to 10 mM KCl for 10 min, followed by 2 h of resting time in artificial cerebral spinal fluid (aCSF). We selected clone#72 for this experiment because of its lower expression of HTTEX1Q72-mCherry, which provided a larger range for an increase in transmission without risk of system saturation. With exposure to 10 mM KCl, we observed a greater number of HTTEx1Q72-mCherry puncta in myotubes than in control co-cultures exposed to 2.5 mM KCl (Figure 5A). As a negative control for HTTEx1Q72-mCherry transmission, we used the Neu mCherry cell line, which does not express HTTEx1Q72. In co-cultures with this cell line, no mCherry puncta were observed in myotubes (Figure 5A). The increase in HTTEx1Q72-mCherry puncta in myotubes with 10 mM KCl treatment was not associated with a reorganization of postsynaptic structures as the proportions of AChR types did not change after KCl treatment (Figure 5B). Because KCl depolarizes myotubes as well as neurons, we repeated the experiment in MFDs and applied 10 mM KCl only to the pre-synaptic side (Figure 5C, upper panel and lower left panel). We found that intracellular Ca^2+^ levels in myotubes were significantly increased when neurons were exposed to 10 mM KCl compared with that when they were exposed to 2.5 mM KCl (Figure 5C, lower right panel). This confirmed that an increase in neuronal depolarization results in enhanced NMJ activity. We again observed an increase in the number of HTTEx1Q72-mCherry puncta in myotubes with the addition of 10 mM KCl (Figures 5D, E). When we compared the volumes of these HTTEx1 Q72-mCherry puncta, we observed a larger contribution of small assemblies in KCl-treated co-cultures than in non-treated co-cultures, again supporting that transmission occurs in the form of small protein complexes (Figure 5F).

The above results obtained with two opposite synaptic manipulations demonstrated that synaptic activity regulates HTTEx1Q72-mCherry transmission and that the pathway of transmission is coupled with synaptic vesicle release.

### 3.8 HTTEx1Q72-mCherry transmission induced and aggravated pathological alterations in myotubes

An important open question in the field of misfolded proteins is whether their transmission can trigger or aggravate the disease-relevant pathology caused by the cell-autonomous presence of the toxic protein. Addressing this will reveal whether toxic protein transmission should be considered a novel disease pathway in neurodegenerative PMDs. To this end, we generated from the MyoD line an isogenic MyoD iPSC line expressing HTTEx1Q72-mCherry (Supplementary Figure 2A), and sought to identify the presence of HD-specific pathological alterations in myotubes in the following neuronal/myotube co-culture combinations: (1) Neu ctr /Myo ctr [no expression of the pathogenic HTTEx1Q72 (**Ctr)**]; (2) Neu ctr/Myo HTTEx1Q72-mCherry [cell-autonomous (**CA)**]; (3) Neu HTTEx1Q72-mCherry/Myo ctr [transmission (**T**)]; and (4) Neu HTTEx1Q72-mCherry/Myo HTTEx1Q72-mCherry (**T+CA**).

**Figure 6:**
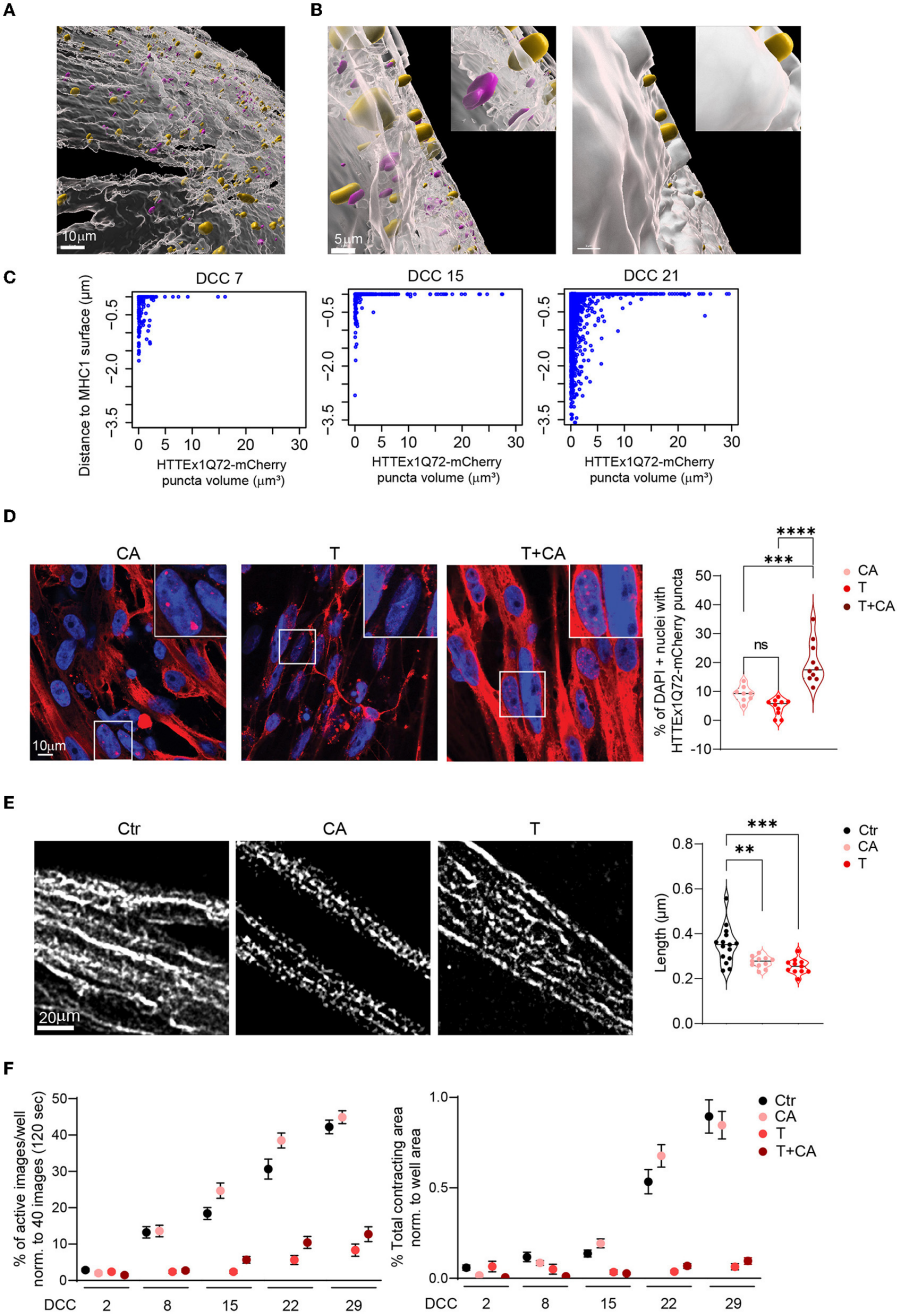
HTTEx1Q72 neuromuscular transmission causes pathological structural and functional alterations in myotubes in a dose-dependent manner. **(A)** Overview image of an Imaris model of a MHC1+ myotube surface (white) with HTTEx1Q72-mCherry puncta associated with (yellow) and inside (magenta) the MHC1+ surface in Neu HTTEx1Q72-mCherry clone#75/myotube co-cultures. (**B**, left panel) Higher magnification of **(A)** shows yellow puncta penetrating the MHC1+ surface and magenta puncta not in contact with the surface. (**B**, right panel) The same image as in “left panel” with the non-transparent MHC1+ surface, in which only the yellow puncta on the outside of the MHC1+ surface are seen. **(C)** Dot plots of the distance from HTTEx1Q72-mCherry puncta to the MHC1+ surface against their volume from DCC 7 to DCC 21. **(D)** IF images of cell-autonomous (**CA**, Neu Ctr mCherry/Myo HTTEx1Q72-mCherry), transmission (**T**, Neu HTTEx1Q72-mCherry/Myo mCherry), and transmission+cell autonomous (**T+CA**, Neu_HTTEx1Q72-mCherry/Myo HTTEx1Q72-mCherry) co-cultures on DCC 21, showing HTTEx1Q72-mCherry labeling in myotube nuclei [labeled with DAPI (blue)]. Bottom right panel: the percentage of myotube nuclei with HTTEx1Q72-mCherry puncta in cultures of the different genotypes (*n* = 10 images/genotype from three independent co-cultures); **p ≤ 0.01, ***p ≤ 0.004, ****p ≤ 0.0001 (one-way ANOVA, Dunnett's correction). **(E)** Images showing the mitochondrial marker TOMM20 in myotubes in control (**Ctr**, Neu mCherry/Myo mCherry), **CA**, and **T** co-cultures. The associated graph shows the quantification of mitochondrial length in these mixed-genotype co-cultures (*n* = 30–40 images/genotype from three independent MFD co-cultures). **(F)** Quantification of myotube contraction parameters measured in **Ctr**, **T**, **CA**, and **T+CA**_co-cultures from DCC 2 to DCC 29 (*n* = 22–27 wells/genotype and time point from three independent co-cultures). Three-way mixed-design ANOVA: Significant transmission-dependent decrease in myotube contraction parameters dependent on time and independent of cell-autonomous expression (*p* = 5.71e-34 left panel, *p* = 2.00e-21 right panel). A significant increase in the percentage of active images in cell-autonomous cultures, dependent on time and independent of transmission (*p* = 0.003). DCC, days of co-culture; MFD, microfluidic device. All averaged data are shown as means ± SEM.

It has been shown that mHTT has a high affinity for bioengineered lipid membranes and that the insertion of these proteins into these membranes triggers their aggregation (Marquette et al., 2020). The close association of the NMJ with the lipid membrane allows to assess whether the transmission of mHTT across the NMJ would lead to mHTT enrichment close to the myotube membrane. We labeled the myotubes with MHC1, a class-I myosin that directly binds to components in the lipid membrane {Pyrpassopoulos, 2016 #196} and used the Surface function of the Imaris software (Oxford Instruments) to define the surface of myotubes based on MHC1 staining. We assessed the proportion and size of HTTEx1Q72-mCherry puncta directly associated with the MHC1+ surface relative to those found at a defined distance from the MHC1+ surface. Specifically, we examined the intracellular localization of HTTEx1Q72 in the myotubes in **T** cultures from DCC 7 to DCC 21, in particular the localization of the HTTEx1Q72-mCherry puncta at the myotube surface. Visual inspection of the obtained images revealed a striking localization of the puncta on and partially traversing the MHC1+ myotube surface (Figures 6A, B). On DCC 7, half of the puncta were localized at the myotube surface (0–0.05 μm to the myotube surface) or inside the myotube (>0.05 μm to the myotube surface), and most were small (< 4 μm^3^) (Figure 6C). On DCC 15, even more (79%) of the HTTEx1Q72-mCherry puncta accumulated at the surface, but on DCC 21, this had shifted back to 52% (Figure 6C). Furthermore, as we found before (Figure 2H), the number of larger HTTEx1Q72-mCherry puncta increased with time (Figure 6C). Our analysis revealed that the largest HTTEx1Q72-mCherry puncta (volume >5 μm^3^) were nearly all (>90%) localized at the myotube surface from DCC 7 to DCC 21 (Figure 6C). These data suggested that mHTT is particularly prone to aggregate close to the lipid membrane, which supports that lipid membranes are, as found for bioengineered lipid membranes, hotspots for mHTT aggregation.

To further assess the pathological consequences of transmitted HTTEx1Q72 for myotubes, we analyzed the extent of nuclear accumulation of HTTEx1Q72-mCherry aggregates in mixed genotype co-cultures. Nuclear aggregates have been detected in skeletal muscle of R6/2 mouse models of HD and their increase was correlated with a worsening of disease pathology (Orth et al., 2003). Here, the lowest number of nuclear aggregates were detected in **T** co-cultures, followed by **CA** co-cultures, which showed a small increase in the number of aggregates, and, finally, **T+CA** co-cultures, in which a significant increase in the number of aggregates was observed compared to **T** and **CA** cultures (Figure 6D; Supplementary Figure 10A).

Next, we analyzed mitochondria, as dysfunction of these organelles is a characteristic observed in brain and skeletal muscle obtained from patients with HD and animal models of the disease (Zielonka et al., 2014; Jurcau and Jurcau, 2023). Typically, there is an imbalance in fission and fusion events, which leads to increased mitochondrial fragmentation and a smaller filamentous network (Reddy, 2014; Kojer et al., 2019). To assess the effect of **CA** and **T** HTTEx1Q72 on mitochondria fission/fusion, we compared mitochondrial length, area weighted form factor (AWFF), and form factor (FF) between **CA** or **T** co-cultures and **Ctr** co-cultures. We used MFDs to avoid contamination with neuronal mitochondria. We did not analyze **T+CA** co-cultures because we would not be able to discriminate myotubes that received HTTEx1Q72-mCherry from neurons from those that did not. A significant reduction in all three parameters was observed in both the **CA** and **T** conditions compared with that in the **Ctr** condition (Figure 6E; Supplementary Figures 10B, C). Fragmentation of the mitochondrial filamentous network is likely to impair mitochondrial function. For example, ATP production is reduced in the skeletal muscles of patients with HD, and patients already exhibit exercise-induced muscle fatigue at preclinical stages of the disease (Lodi et al., 2000; Ciammola et al., 2011). To assess whether any functional change occurred in myotubes, we measured myotube contractions. Strikingly, we observed a nearly complete loss of myotube contractions selectively in **T** or **T+CA** co-cultures as assessed by the measurement of both activity and contraction area (Figure 6F).

## 4 Discussion

Whether cell-to-cell transmission of mHTTEx1 is regulated by functional synaptic connectivity and can contribute to disease pathology in an environment where the mutant protein is ubiquitously expressed is not well-understood. To advance the current understanding of these processes, we established two *in vitro* hiPSC-derived neuromuscular co-culture systems to study the role of synaptic connections in the development of HD-related pathology. We provide evidence that neuromuscular transmission of mHTTEx1 can occur across the human neuromuscular synapse and contributes to skeletal muscle pathology. Furthermore, our results suggested that there is a positive correlation between synaptic activity and mHTTEx1 transmission and between synaptic density and mHTTEx1 transmission.

With the human neuromuscular co-culture system established in this work, we could generate functional NMJs both in on-top co-cultures and in MFDs, which permitted us to deepen our understanding of transsynaptic mHTT transmission. Employing the Cre-Lox system, we could follow HTTEx1Q72-Cre transmission in the same culture over weeks using fluorescent live-cell imaging. The presence of GFP expression in Myo LoxP-GFP cells demonstrated that HTTEx1Q72-Cre is transmitted from neurons to muscles and can reach the nucleus of myotubes. The N-terminal fragment of mHTT, which contains exon 1, is known to translocate to the nucleus, where it induces the dysregulation of transcriptional processes and the mislocalization of the nuclear pore complex (Kumar et al., 2014; Grima et al., 2017).

MHTT aggregation is a concentration-dependent process. The expression level of the mutant protein in HTTEx1Q72-Cre neurons was sufficiently low to allow us to investigate mHTT transmission before aggregation occurred. We demonstrated that transmission occurred over weeks in the absence of detectable mEM48+ aggregates in neurons. Aggregates were first detected in myotubes on DCC 28. Using Neu HTTEx1Q72-mCherry/Myo LoxP-GFP co-cultures, we confirmed that transmission resulted in an increase in the abundance of predominantly small protein assemblies in the myotubes over time and that larger protein structures formed with increasing culture duration. This finding is supported by a recent study by Miguez et al. (2023), in which the authors showed that neuronal cells derived from patients with HD transplanted to the striatum of healthy mice transmitted soluble monomeric and/or oligomeric mHTT through exosomes to their synaptic target regions. The same authors also showed that the formation of mHTT aggregates followed the appearance of the soluble form of the protein. Unlike insoluble aggregates such as fibrils or inclusion bodies, monomeric and oligomeric structures, which are soluble, can move more easily and are thus more likely to be transmitted between cells (Herrera et al., 2011; Miguez et al., 2023). This suggests that mHTT transmission likely occurs in monomeric/oligomeric form and progressively leads to aggregate pathology.

Our findings indicated that MHTTEx1 transmission was dependent on direct neuron–myotube connectivity and occurred already during NMJ assembly. HTTEx1Q72-mCherry puncta were associated with NMJ-forming AChR cluster types and transmission was positively correlated with NMJ density. It has been shown that neuron-to-neuron tau transmission is enhanced with increasing synaptic density (Calafate et al., 2015). These data suggest that toxic protein spreading may represent a very early and progressive pathological process in HD and support that connectivity *per se* and the density of synaptic connectivity between cells might be important factors influencing toxic protein levels in postsynaptic cells (Poudel et al., 2019).

Recent work has demonstrated that mHTTEx1 secretion is a regulated process (Tang, 2018). Additionally, several mechanisms of unconventional mHTT secretion have been described. One involves GRASP55, a Golgi reassembly stacking protein that regulates unconventional, Golgi-independent secretion of cytosolic and transmembrane cargoes. It has been shown that mHTT is secreted in a GRASP55 and autophagy-dependent manner and that this secretion is affected by stress conditions (Ahat et al., 2022). Additionally, the Rhes protein has been demonstrated to transport mHTT across tunneling nanotube-like structures between neurons derived from patients with HD and mouse striatal medium spiny neurons as well as from striatal to cortical regions *in vivo* in mice (Ramirez-Jarquin et al., 2022). In our study, we found that HTTEx1Q72-mCherry transmission was enhanced by neuronal depolarization and decreased when neuronal presynaptic release was blocked. This observation, together with previous studies on Aβ, tau, and mHTTEx1, strongly suggests that transmission occurs across functional synapses and is enhanced by presynaptic activity (Cirrito et al., 2005; Pecho-Vrieseling et al., 2014; Babcock and Ganetzky, 2015; Calafate et al., 2015; Caron et al., 2021). In contrast, in *Drosophila*, the transsynaptic transmission of mHTT is inversely correlated with neuronal activity and depends on phagocytic glia (Donnelly et al., 2020). This difference might be explained by the use of different mHTT constructs; alternatively, they may reflect different cellular mechanisms of spreading. For instance, transneuronal spreading of misfolded proteins has been shown to occur through extracellular vesicles and exocytosis/endocytosis (Jeon et al., 2016; Zhang et al., 2016; Trajkovic et al., 2017; Miguez et al., 2023), processes that are regulated by neuronal activity. Before neuronal damage becomes apparent, neuronal activity could play an important role in transsynaptic transmission, whereas transsynaptic transmission by phagocytic glial cells could be important as synaptic activity progressively decreases as a result of cell damage. In addition to a non-constitutive pathway of secretion, constitutive secretion is supported by our observation that hiPSC clones with higher HTTEx1Q72 expression levels showed greater levels of mHTT transmission to muscle [~15-fold increase in the number of HTTEx1Q72-mCherry puncta between myotubes cultured with clones #72 (low-expressing; Figure 5A) and #75 (high-expressing; Figure 4B)]. Similarly, positive correlations have been reported to exist between the intracellular levels of α-syn and Aβ and the amounts of the respective proteins that are released (Domert et al., 2014; Reyes et al., 2015). Regulated and constitutive release might go hand-in-hand. For instance, the intracellular presence of misfolded proteins might trigger a highly sensitive stress response, resulting in active transmission of the toxic species.

We have also demonstrated that chronic inhibition of neurotransmitter release via the exposure of neuromuscular co-cultures to TeNT resulted in aggravated nuclear aggregate-related pathology in HTTEx1Q72-mCherry-expressing neurons. Similarly, mHTT secretion is inhibited in *Grasp55* knockout mice, and this also results in enhanced HTT aggregation and toxicity (Ahat et al., 2022). The clearance of misfolded proteins by the ubiquitin–proteasome system and autophagy is crucial for preventing protein accumulation and, consequently, aggregation (Ciechanover and Kwon, 2015; Zhao et al., 2016). Our findings suggest that toxic protein release might represent an as-yet undefined pathway for misfolded protein clearance. A similar observation has been made for Aβ (Domert et al., 2014). Accordingly, novel drug discovery strategies should focus on promoting the release and preventing the uptake of mHTT. Current antibody-based therapies designed to prevent Aβ, tau, and α-syn accumulation in tau- and synucleinopathies also merit testing in HD (Valera and Masliah, 2013; Plotkin and Cashman, 2020).

It has been shown that transmission alone induces non-cell autonomous pathology in hiPSC-derived neurons, as well as *in vivo* in *Drosophila* and *C. elegans* (Pecho-Vrieseling et al., 2014; Babcock and Ganetzky, 2015). Furthermore, the expression of mHTTEx1 in selective brain regions *in vivo* in mice and non-human primates results in the propagation of the transgenic protein to other, non-expressing brain regions and can induce motor deficits and cognitive decline after weeks/months (mice) or years (non-human primates) as well as cell death (Masnata et al., 2019; Maxan et al., 2020; Miguez et al., 2023). However, the pathobiological relevance of misfolded protein transmission in an environment in which a toxic protein is ubiquitously expressed, as occurs in HD, has not been assessed to date. Here, we demonstrated the existence of a causal link between transmission and pathology. Using a mixed-genotype co-culture system, we dissected the contribution of transmission to cell-autonomous pathology. Mitochondrial fragmentation and accumulation of intranuclear mHTT aggregates are two pathological hallmarks of HD in muscle. In our study, we found that these pathologies occurred in myotubes both when mHTT was expressed cell-autonomously and when it was transmitted from neurons. Importantly, intranuclear aggregations were exacerbated when the myotubes expressed mHTT cell-autonomously and additionally received mHTT via transmission from motor neurons. Given the complexity of mitochondrial physiology, the mechanisms underlying mitochondrial dysfunction in our cellular system (including the assessment of the expression of proteins involved in mitochondrial fission/fusion, mitochondrial respiration, and ATP production) require further study. Several therapeutic strategies that aim to lower mHTT protein levels have already been tested, and have the potential to counteract these pathologies.

mHTT has a strong affinity for lipid membranes and bioengineered lipid bilayers have been shown to function as mHTTEx1 aggregate-promoting structures (Suopanki et al., 2006; Atwal et al., 2007; Trevino et al., 2012; Burke et al., 2013; Gao et al., 2016; Tao et al., 2019). In line with these findings, we detected a temporal increase in the numbers of the larger HTTEx1Q72-mCherry puncta, which preferentially localized to the myotube surface. This suggested that aggregation occurs at the myotube membrane. mHTT aggregation in the membrane is likely to disrupt the lipid bilayer and potentially lead to the distorted localization of membrane receptors, including those required for normal transsynaptic signaling (Iuliano et al., 2021). Interestingly, we found that the transmission of mHTTEx1 from neurons to myotubes induced a marked decrease in myotube contractibility, which was not observed when the mutant protein was expressed exclusively in myotubes. Synaptic dysfunction is an early pathological phenomenon of HD (Barron et al., 2021). The regulated release and uptake of mHTTEx1 might result in its enrichment at synaptic sites and contribute to this pathology. Alternatively, mitochondrial pathology may induce synaptic dysfunction and, consequently, a pronounced decrease in myotube contractibility (Sawant et al., 2021). Whether the above factors contribute to the transmission-selective loss of myotube contractions needs further investigation. Regarding the observed pathologies, it would be highly interesting in future studies to assess the link between mHTT transmission and muscle atrophy.

Taken together, the positive correlation observed among NMJ density, the abundance of HTTEx1-mCherry puncta, and transmission-triggered pathology suggests that the existence of a large number of synaptic connections in the CNS and between spinal motor neurons and skeletal muscle renders these regions particularly susceptible to HD. In this context, it is interesting that striatal medium spiny neurons are the most vulnerable cell types in HD. They receive a large number of synaptic inputs from cortical, subcortical, and intra-striatal neurons, and have intrinsically silent activity patterns and low excitability (Gerfen and Surmeier, 2011). These properties might make them particularly susceptible to mHTT accumulation due to a potentially high-receive, low-release situation. Furthermore, mHTT accumulation is likely to be particularly devastating for medium-sized spiny neurons because mHTT greatly impairs mitochondrial function and medium-sized spiny neurons are strongly dependent on mitochondrial function for their survival, possibly because they require large amounts of ATP to maintain a hyperpolarized state (Han et al., 2010).

Given the peripheral phenotype, our findings provide novel opportunities for therapeutic and biomarker development for target regions accessible to interventions and disease progression assessment.

In a broader context, the transsynaptic transmission of misfolded proteins is likely to be a common mechanism in PMDs by which these toxic species spread through the brain and the periphery, contributing to a temporal decline in the functional abilities of patients.

## Data availability statement

The original contributions presented in the study are included in the article/Supplementary material, further inquiries can be directed to the corresponding author.

## Ethics statement

Ethical approval was not required for the studies on humans in accordance with the local legislation and institutional requirements because only commercially available established cell lines were used.

## Author contributions

MD: Conceptualization, Data curation, Formal analysis, Investigation, Methodology, Validation, Visualization, Writing – review & editing, Project administration. LC: Conceptualization, Data curation, Formal analysis, Investigation, Methodology, Project administration, Validation, Visualization, Software, Writing – original draft. UB: Formal analysis, Investigation, Methodology, Software, Validation, Visualization, Writing – original draft. AG: Formal analysis, Investigation, Methodology, Validation, Visualization, Writing – original draft. IF: Formal analysis, Investigation, Methodology, Validation, Writing – original draft. IH: Data curation, Formal analysis, Investigation, Methodology, Software, Writing – original draft. DG: Data curation, Formal analysis, Investigation, Methodology, Writing – original draft. AE: Methodology, Resources, Supervision, Writing – review & editing. MM: Methodology, Resources, Supervision, Writing – review & editing. EP-V: Conceptualization, Data curation, Formal analysis, Funding acquisition, Investigation, Methodology, Resources, Supervision, Validation, Visualization, Writing – review & editing.
