# Supplementary material for: Transmission-selective muscle pathology induced by the active propagation of mutant huntingtin across the human neuromuscular synapse
Source: Front Mol Neurosci. 2024 Jan 3;16:1287510. doi: 10.3389/fnmol.2023.1287510 (PMC10791992; doi:10.3389/fnmol.2023.1287510)
Supplement: Supplementary file 1 [file Data_Sheet_1.PDF]

A

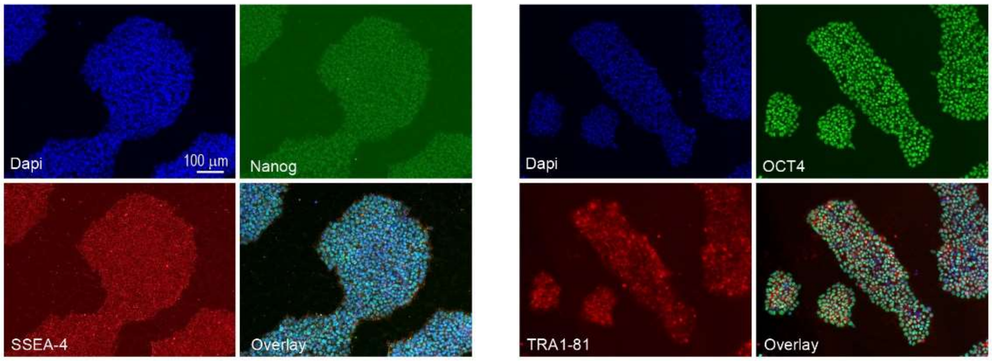

B

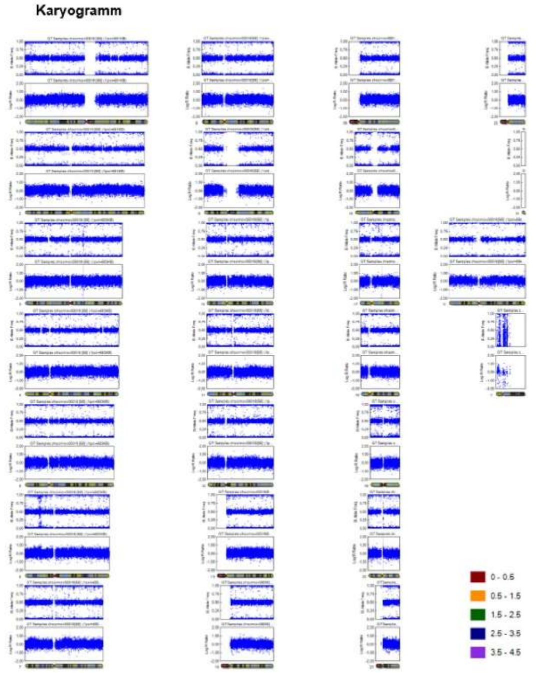

C

Experiment Filename		20141217scorecard3eds.eds	20141217scorecard3eds.eds
Target Name	Category	IPS HDfa90/1.2	EBs HDfa90/1.2
ACTB	Controls	17.59	18.98
ACTB	Controls	22.67	22.11
ACTB	Controls	23.60	23.12
ACTB	Controls	17.84	18.52
CTCF	Controls	25.09	25.12
EP300	Controls	25.91	26.37
SMAD1	Controls	26.66	26.22
CDH9	Ectoderm	32.50	30.78
COL2A1	Ectoderm	30.79	25.14
DMBX1	Ectoderm	30.90	29.48
DRD4	Ectoderm	28.55	26.87
EN1	Ectoderm	40.00	32.00
LMX1A	Ectoderm	35.03	28.78
MAP2	Ectoderm	30.49	26.15
MYO3B	Ectoderm	35.02	29.93
NOS2	Ectoderm	31.37	24.98
NR2F1/NR2F2	Ectoderm	39.65	23.98
NR2F2	Ectoderm	40.00	26.57
OLFM3	Ectoderm	32.31	28.37
PAPLN	Ectoderm	29.40	30.20
PAX3	Ectoderm	38.49	28.01
PAX6	Ectoderm	36.57	23.52
POU4F1	Ectoderm	35.26	27.33
PRKCA	Ectoderm	26.88	27.95
SDC2	Ectoderm	25.55	25.47
SOX1	Ectoderm	34.42	32.46
TRPM8	Ectoderm	33.31	27.97
WNT1	Ectoderm	40.00	28.89
ZBTB16	Ectoderm	35.38	25.01
AFP	Endoderm	40.00	28.31
CABP7	Endoderm	30.32	31.57
CDH20	Endoderm	32.96	26.63
CLDN1	Endoderm	30.64	26.78
CPLX2	Endoderm	30.28	29.77
FLAVL3	Endoderm	28.48	29.33
EOMES	Endoderm	35.14	30.60
FOXA1	Endoderm	39.71	31.33
FOXA2	Endoderm	35.37	32.11
FOXP2	Endoderm	33.74	30.41
GATA4	Endoderm	35.13	29.25
GATA6	Endoderm	33.48	27.68
HHEX	Endoderm	28.41	29.93
HMP19	Endoderm	34.16	33.54
HNF1B	Endoderm	35.97	32.77
HNF4A	Endoderm	35.40	30.59
KLF5	Endoderm	30.66	29.41
LEFTY1	Endoderm	29.75	33.97
LEFTY2	Endoderm	30.45	29.90
NODAL	Endoderm	30.38	28.91
PHOX2B	Endoderm	40.00	32.47
POU3F3	Endoderm	33.51	30.55
PRDM1	Endoderm	31.14	28.34
RXRG	Endoderm	40.00	28.98
SOX17	Endoderm	40.00	32.81
SST	Endoderm	34.99	24.63
FGF4	Mesendoderm	27.29	29.66
GDF3	Mesendoderm	28.28	30.17
NPPB	Mesendoderm	35.33	29.42
NRSA2	Mesendoderm	29.04	31.95
PTHLH	Mesendoderm	35.45	32.61
T	Mesendoderm	37.30	34.01
ABCA4	Mesoderm	35.07	28.64
ALOX15	Mesoderm	30.11	26.78
BMP10	Mesoderm	40.00	34.32
CDH5	Mesoderm	38.09	30.87
CDX2	Mesoderm	40.00	29.34
COLEC10	Mesoderm	34.25	35.14
ESM1	Mesoderm	40.00	31.16
FCN3	Mesoderm	32.08	32.10
FOXF1	Mesoderm	35.57	28.85
HAND1	Mesoderm	31.70	24.86
HAND2	Mesoderm	40.00	27.31
HEY1	Mesoderm	31.72	27.97
HOPX	Mesoderm	35.11	30.37
IL6ST	Mesoderm	29.18	27.08
NKX2-5	Mesoderm	36.82	34.92
ODAM	Mesoderm	39.58	31.05
PDGFRA	Mesoderm	30.82	28.66
PLVAP	Mesoderm	32.24	29.29
RGS4	Mesoderm	34.94	27.23
SNAI2	Mesoderm	34.00	27.06
TBX3	Mesoderm	34.11	26.21
TM4SF1	Mesoderm	30.26	31.67
CD44	Other	40.00	40.00
JARID2	Other	23.28	24.99
MYC	Other	26.71	26.43
SEV	Other	40.00	40.00
CXCL5	Self-renewal	26.65	32.60
DNMT3B	Self-renewal	19.76	22.65
HESX1	Self-renewal	27.08	27.66
IDO1	Self-renewal	24.60	31.26
LCK	Self-renewal	27.28	31.24
NANOG	Self-renewal	24.03	27.37
POU5F1	Self-renewal	28.83	32.64
SOX2	Self-renewal	22.17	22.69
TRIM22	Self-renewal	25.56	29.83

Sample Name	Self-renewal	Ectoderm	Mesoderm	Endoderm
IPS hDfa90/1.2	POSITIVE	NEGATIVE	NEGATIVE	NEGATIVE
EBs hDfa90/1.2	NEGATIVE	POSITIVE	POSITIVE	POSITIVE

Supplementary Figure 1. The characterization of the hiPSC line hDFa90/1.2. **(A)** IF staining of the hDFa90/1.2 hiPSCs with antibodies against the pluripotency markers Nanog, SSEA-4, OCT-4, and TRA1-81. **(B)** Karyogram of the hDFa90/1.2 hiPSC line performed by full-genome SNP analysis. **(C)** Scorecard of the hDFa90/1.2 hiPSC line and embroid bodies (EBs) differentiated from the hDFa90/1.2 hiPSC line.

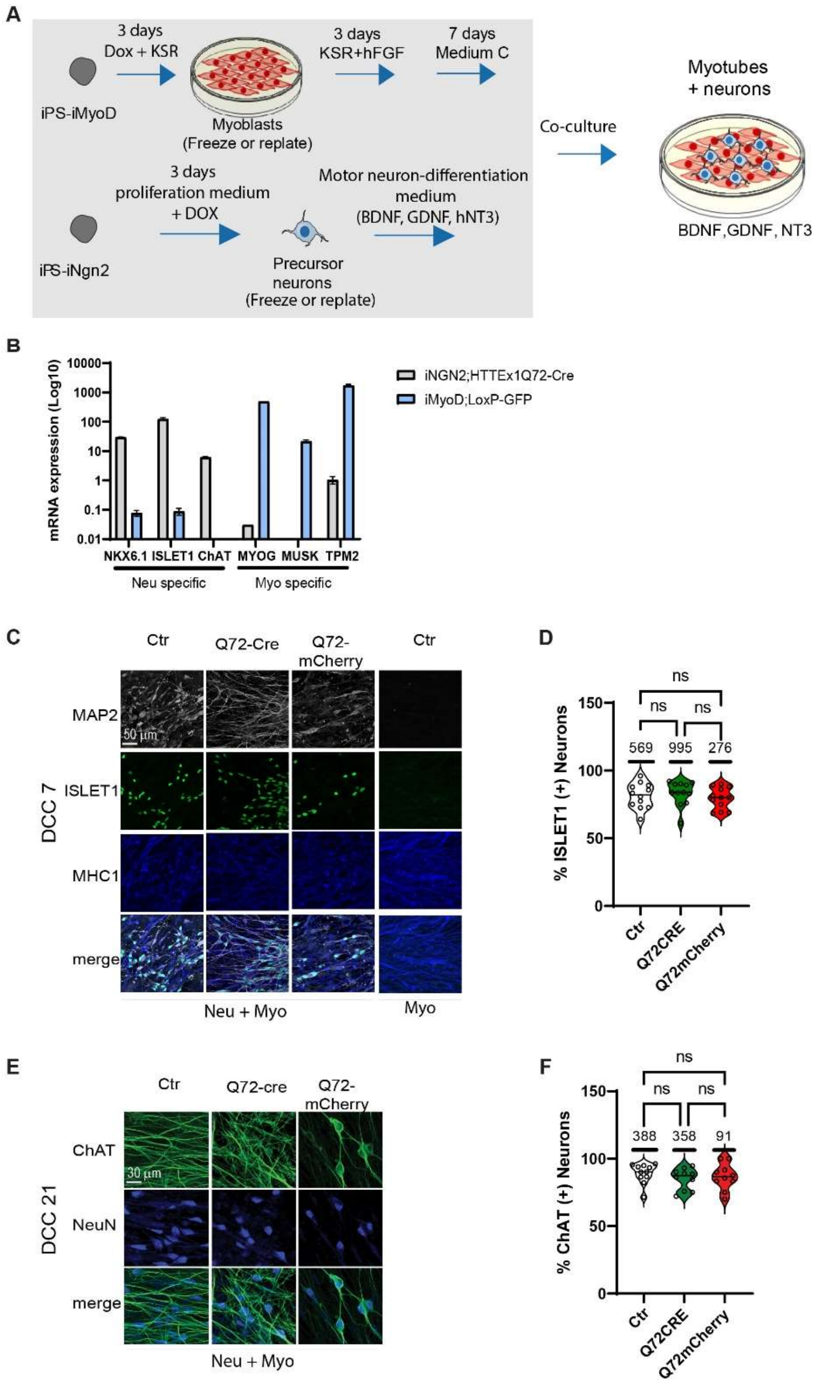

Supplementary Figure 2. Selective expression of myotube- and neuron-specific mRNA and protein markers in iMyoD;FloxP-GFP and iNGN2;HTTEX1-Cre, respectively. (**A**) Diagram showing the 2-step protocol used to establish the neuromuscular co-culture. (**Grey box**) iMyoD and iNgn2 hiPSCs are cultured and differentiated by exposing them for 3 days to DOX, into myoblasts and precursor neurons, resp. Myoblasts are frozen at this stage or seeded (replated) and cultured in myotube-promoting media to allow further differentiation and maturation of the myoblasts into myotubes. Precursor neurons are frozen or seeded and cultured in motor neuron differentiation medium containing BDNF, GDNF and hNT3 to promote differentiation and further maturation into motor neurons. (**Right panel**) Neuromuscular co-cultures are established 10 days after the seeding of the myoblasts. The precursor neurons are seeded on top of the myotubes. This we indicate as DCC 0. The co-culture is grown in motor neuron-promoting media. (**B**) Graph depicting the mRNA expression levels of motor neuron- and myotube-specific mRNAs in iNGN2 motor neurons (grey bars) and iMyoD myotubes (blue bars). (**C**) IF labeling of iNGN2 motor neurons + iMyoD myotube co-cultures with the following motor neuron lines: Ctr (iNGN2 line w/o expression of HTTEx1Q72 and w/o expression of tag), Q72-Cre (iNGN2 expressing HTTEx1Q72-Cre), Q72-mCherry (iNGN2 expressing HTTEx1Q72-mCherry) with antibodies against motor neuron (MAP2, ISLET1)- and myotube (MHC1)-specific markers. Panel on the right depicts monoculture of iMyoD

myotubes. (**D**) Quantification of % ISLET1+ neurons normalized to MAP2+ neurons. Ctr: mean+/-SEM = 81% +/- 3%; Q72Cre: 83% +/- 3%; Q72mCherry: 80% +/- 2% (n=12 images from n=3 independent experiments. The numbers above each column indicates the number of cells analyzed. Significance was tested with ordinary one-way ANOVA) (**E**) IF labeling of co-cultures with motor neuron-specific antibodies. (**F**) Quantification of % ChaT positive neurons normalized to NeuN+ neurons. Ctr: mean+/-SEM = 89% +/- 2%; Q72Cre: 85% +/- 3%; Q72mCherry: 87% +/- 3% (n=12 images from n=3 independent experiments. The numbers above each column indicates the number of cells analyzed, Significance was tested with ordinary one-way ANOVA) . Abbreviations: **BDNF** = brain-derived neurotrophic factor; **DCC** = day of co-culture; **DOX** = doxycycline; **GDNF** = glial cell line-derived neurotrophic factor; **hNT3** = human neurotrophin 3; **IF** = immunofluorescence; **Myo** = myotube; **Neu** = neuron.

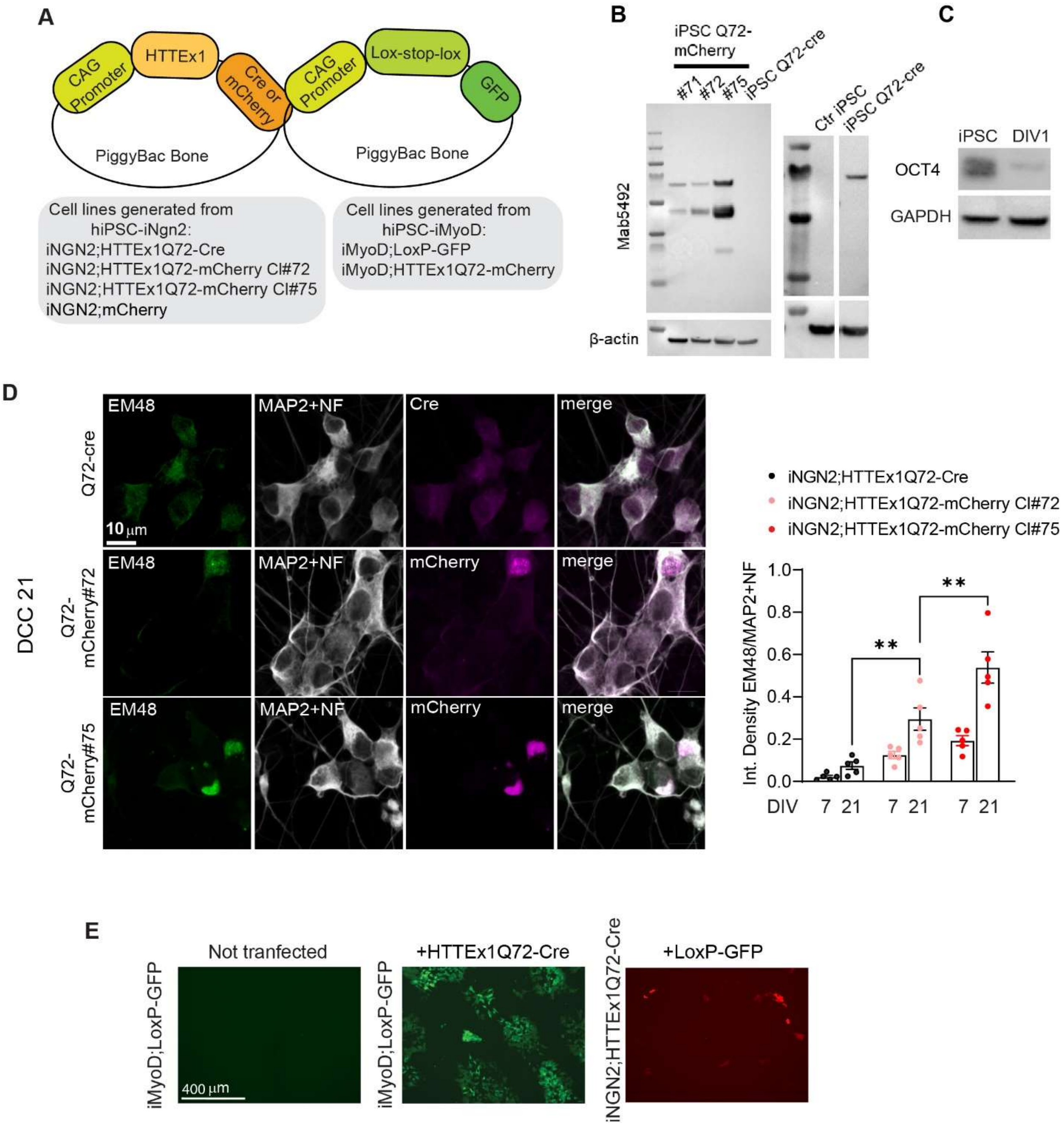

Supplementary Figure 3. An overview of the different hiPSC-derived iNGN2 and iMyoD lines generated and their characterization. (**A, left panel**) Schematic of a plasmid map used for generating the four pro-neuronal HTTEx1 expressing iNGN2 hiPSC lines. (**A, right panel**) Schematic of a plasmid map used for generating the two pro-muscle iMyoD hiPSC lines. The iMyoD line is isogenic to the iNGN2 line. (**B**) WB of HTTEx1Q72 expression visualized with Mab5492 Ab in hiPSCs iNGN2;HTTEx1Q72-mCherry clones: 71, 72 and 75. To visualize the expression of HTTEx1Q72 in the hiPSC HTTEx1-Q72-Cre line exposure time of the western blot had to be increased (right). (n=3) (**C**) Representative WB of pluripotent marker OCT4 in iNGN2; HTTEx1Q72-Cre hiPSCs and

precursor neurons at DIV 1. (**D, left panel**) Representative IF images of the quantification shown in the bar graph to the right, visualizing EM48 and MAP2+NF staining in iNGN2;HTTEx1Q72-Cre, iNGN2;HTTEx1Q72-mCherry clone #72 and, iNGN2;HTTEx1Q72-mCherry clone #75 (**D, right panel**) Quantification of EM48 fluorescent intensity in neurons at DIV 7 and 21, **p<0.01, ***p<0.0001 (5 pictures from 3 independent cultures, one-way ANOVA, Tukey's correction). (**E**) IF images visualizing absence of GFP expression in iMyo;LoxP-GFP hiPSCs not transfected with HTTExQ72-Cre plasmid (**left image**) and presence of GFP expression for the cells transfected with HTTExQ72-Cre plasmid (**middle image**) and expression of mCherry when the hiPSC iNgn2;HTTEx1-Cre are transfected with a LoxP-mCherry plasmid (**right image**). Abbreviations: **DIV** = day in vitro; **IF** = immunofluorescence; **Int. density** = integrated density; **WB**= western blot. All averaged data are shown as the mean ± SEM.

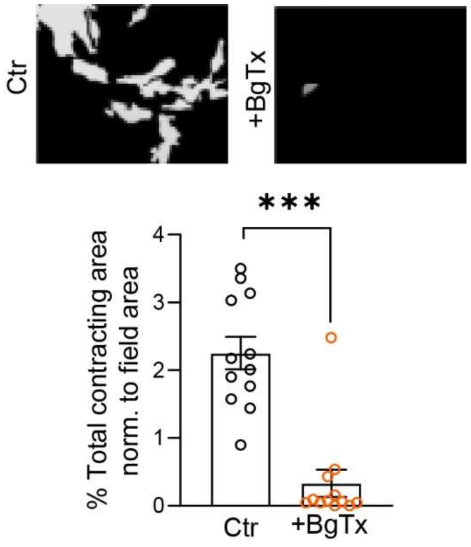

Supplementary Figure 4. Treatment of neuromuscular cultures with BgTx results in the absence of myotube contractions. **(Top panel**) Images showing contracting area of myotubes in Neu HTTEx1Q72-Cre/Myo LoxP-GFP cultures before and after treatment with 2.5 µM BgTx at DCC 22. **(Bottom panel**) Quantification of the percentage total contracting area normalized to well area at DCC 22, before and after treatment with BgTx *** = p=0.0005 (Wilcoxon matched-pairs signed rank test). Abbreviations: **ms** = milliseconds; **BgTx** = α-bungarotoxin (labels AChRs); **DCC**: day of co-culture. All averaged data are shown as the mean ± SEM.

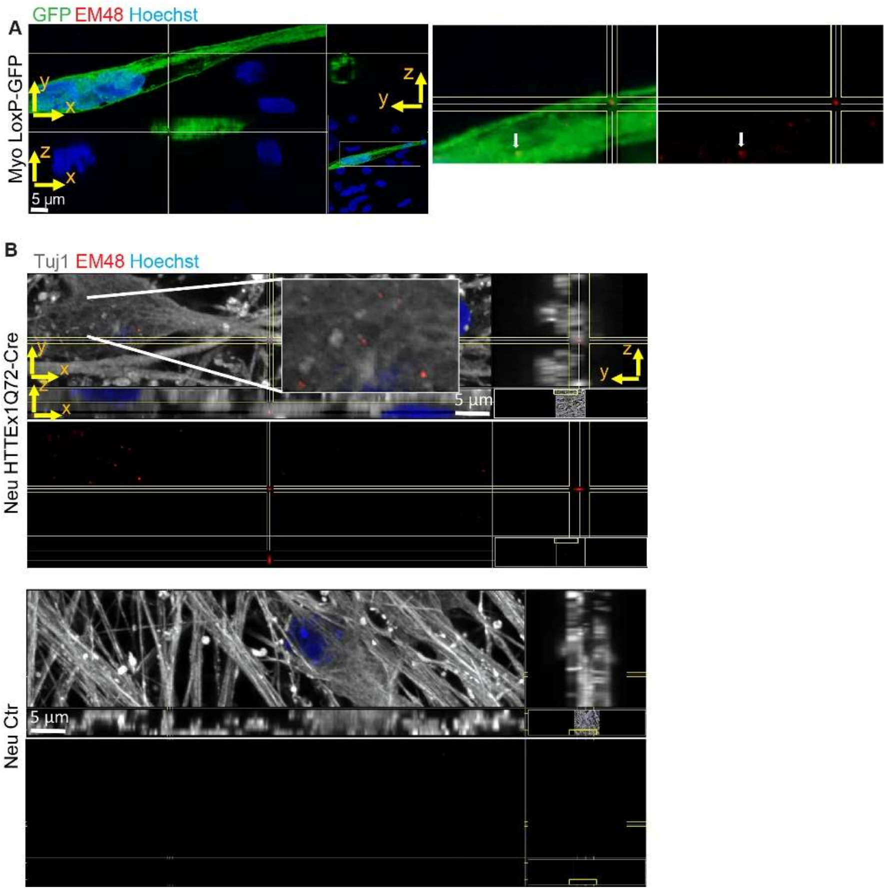

Supplementary Figure 5. EM48+ aggregates in GFP expressing Myo LoxP-GFP myotubes and in Neu HTTEx1Q72-Cre neurons. (**A**) IF staining of Neu HTTEx1Q72-Cre/Myo Lox-GFP co-culture, depicting a GFP+ myotube with EM48+ aggregate at DCC 28. (**B**) IF staining of HTTEx1Q72-Cre and Control neurons at DIV 28, with antibodies against Tuj1 (pan-neuronal marker) and EM48. Abbreviations: **DCC** = day of co-culture; **IF** = immunofluorescence; **Myo** = myotube; **Neu** = neuron.

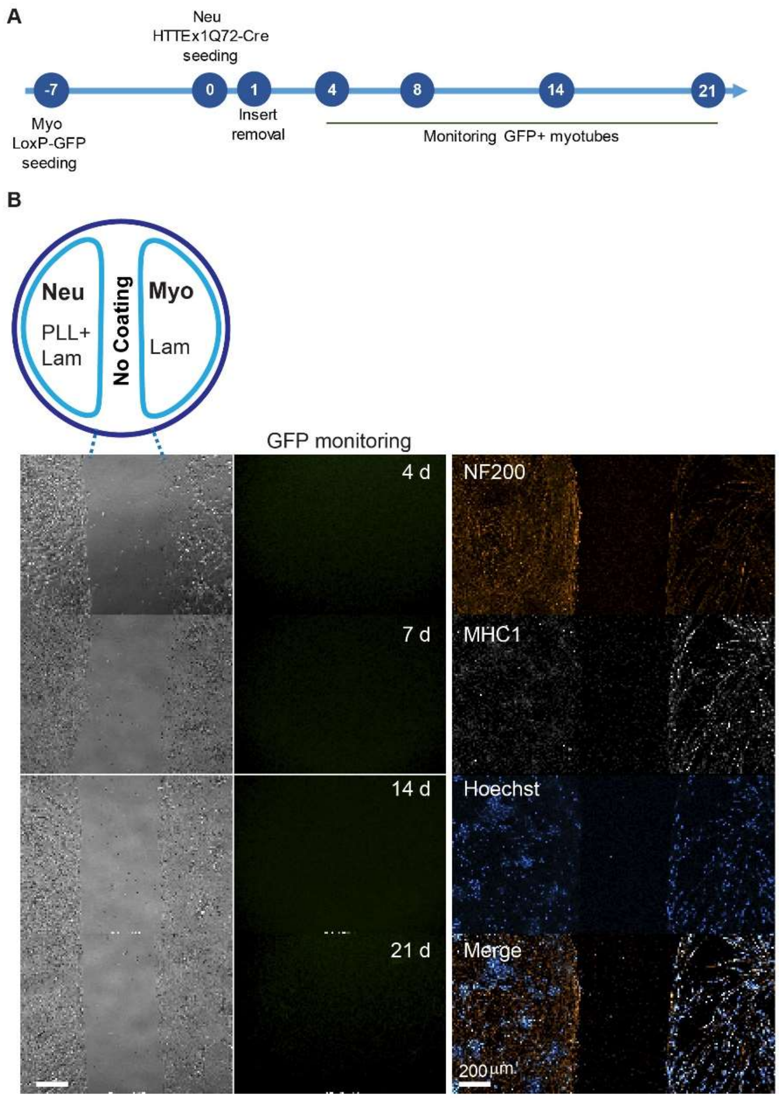

Supplementary Figure 6. Lack of transmission of HTTEx1Q72-Cre from neurons to myotubes in the absence of neuromuscular junctions. (**A**) Timeline of the co-culture experiment in which Neu HTTEx1Q72-Cre and Myo LoxP-GFP were seeded in separate inserts placed in the same culture well. This avoided physical contact between the two cell lines, while it ensured that the two cell lines were exposed to the same culture medium. **(B, top schematic)** Depicting 1 well with the inserts. The insert with neurons is coated with PLLand Lam, the one with myotubes is coated with only Lam. The space between the inserts is not coated to avoid movement and attachment of the cells to this region. (**B, bottom, left panels**) Bright-field image showing physical separation of Neu HTTEx1Q72-Cre and Myo LoxP-GFP cells. (**B, bottom, middle panels**) Showingthe absence of GFP+ myotubes in these cultures at DCC 4 - 21 (n=4 independent devices). (**B, bottom, right panel**) IF images to show expression of the neuron-specific marker NF200, myotube-specific marker MHC1 and the nuclear marker Hoechst. Abbreviations: **PLL** = Poly-L-Lysine; **Lam** = Laminin.

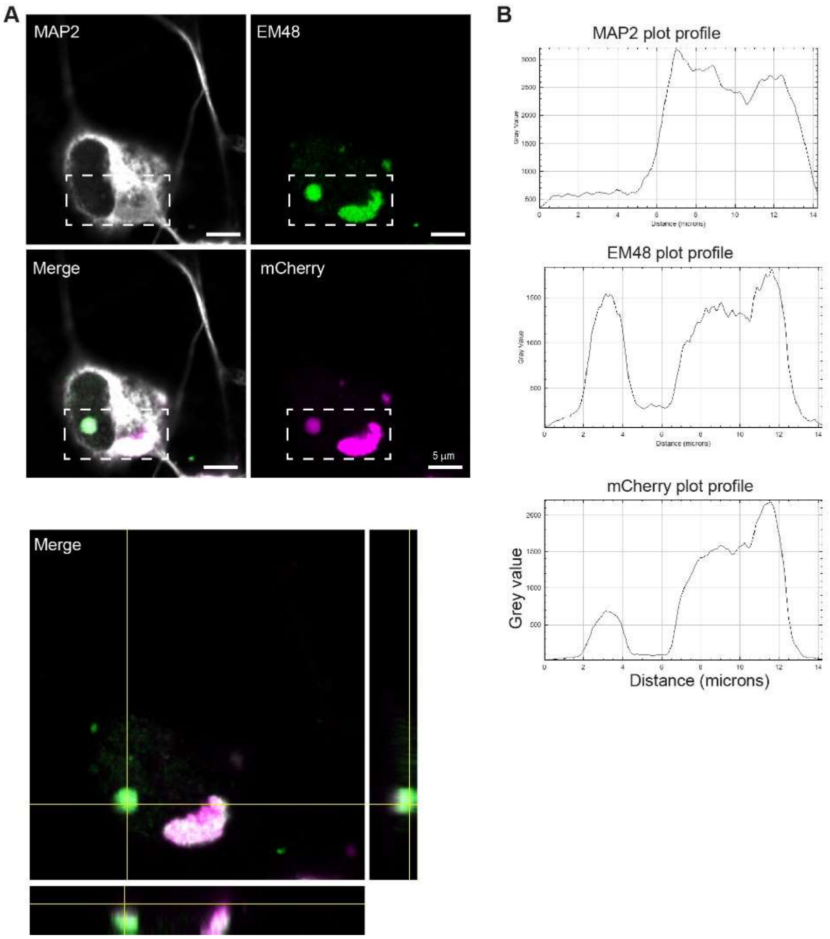

Supplementary Figure 7. Co-localization of EM48 and mCherry in Neu HTTEx1Q72-mCherry neurons. (**A**) IF labeling of HTTEx1Q72-mCherry clone#75 with antibodies against EM48 and mCherry. The lower image shows an orthogonal view of the upper image. **(B)** a plot profile of the individual images displaying the intensities of pixels along a line within the image generated with ImageJ software.

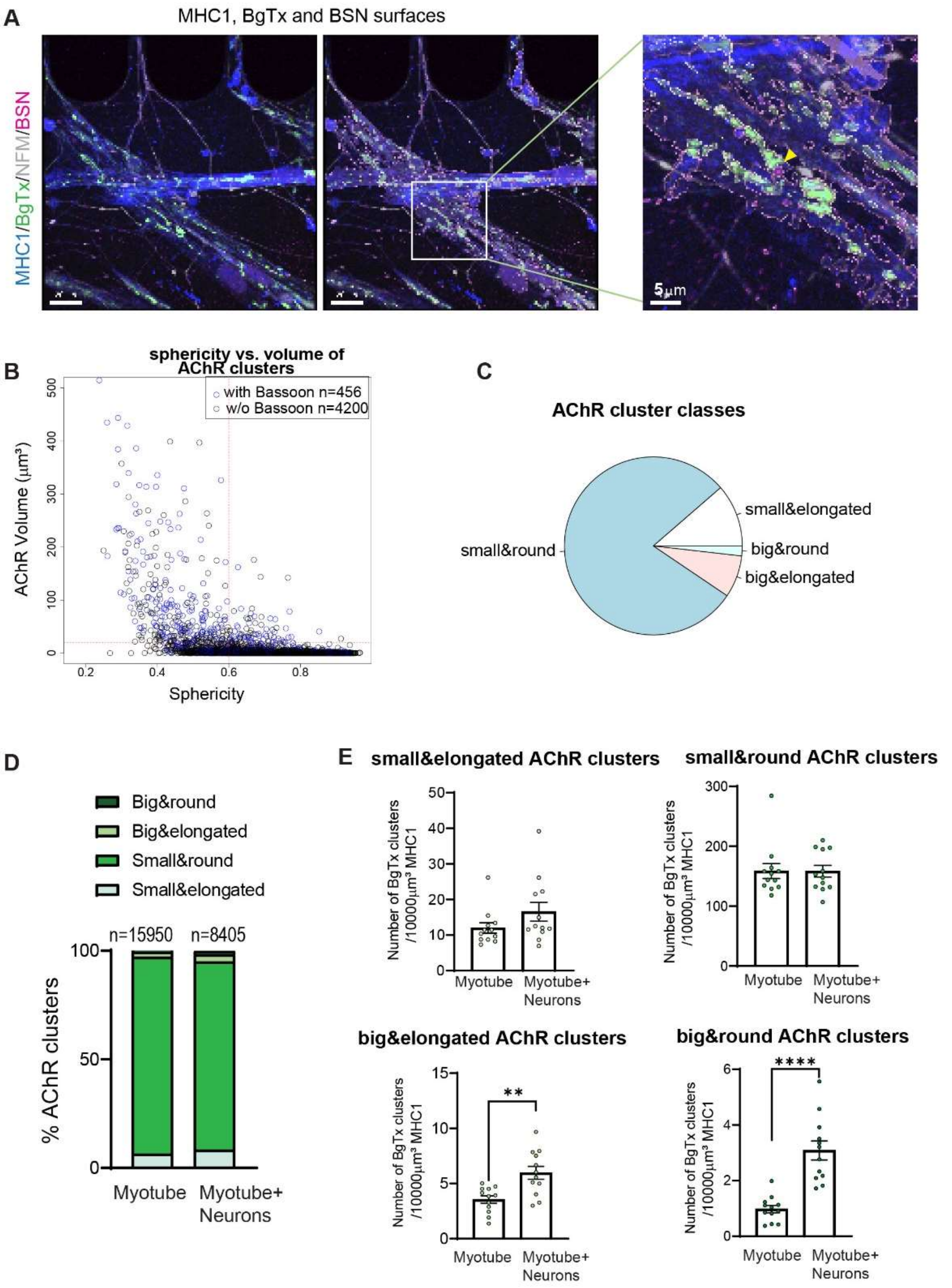

Supplemental Figure 8. Characterization of NMJs in neuromuscular co-cultures. (**A, left image**) IF labeling of neuromuscular co-culture in MFD to visualize the NMJs (appositions between α-BgTx and BSN) between Neu HTTEx1Q72-mCherry cl#75 and Myo LoxP-GFP. The Image is taken from the myotube compartment. (**A, middle image**) Same image as in left image, but with transparent surfaces for MHC1, AChRs and BSN. The surfaces were created with the 'surface' function in Imaris. (**A, right image**) Zoom-in of middle image to visualize the AChR clusters, and apposition of an AChR cluster with BSN (yellow arrowhead). (**B)** Volume against sphericity of the AChR clusters based on

Imaris surface measurements. The red dotted lines indicate the thresholds (20 μm^3 for the volume and 0.6 for the sphericity) used to define four classes of NMJ clusters . Data points for clusters with close appositions between AChRs and BSN (<0.05 μm distance between the surfaces) are colored in blue. (**C**) Pie chart of the proportions of four cluster classes based on data in panel (**B**) (Data from 12 images of Neu HTTEx1Q72-mCherry cl#72 and Myo LoxP-GFP co-cultures in MFD). (**D**) Distribution of four AChR cluster types in percentage, found on myotubes when cultured with or without neurons. (**E**) Comparison of the numbers of AChR clusters in four classes found on myotubes when cultured with or without neurons. The numbers were normalized to myotube volume (based of MHC1 surface). One data point corresponds to one image (12 images were analyzed per condition). ** = $p \leq 0.01$; **** = $p < 0.0001$ (Student's t-test) Abbreviations: **AChRs** = acetylcholine receptors; **BgTx** = α-bungarotoxin (labels AChRs); **BSN** = Bassoon; **DCC** = days of co-culture; **IF** = immunofluorescence; **MHC** = myosin heavy chain, **NMJs** = neuromuscular junctions; **MFD**= microfluid devices. All averaged data are shown as the mean ± SEM.

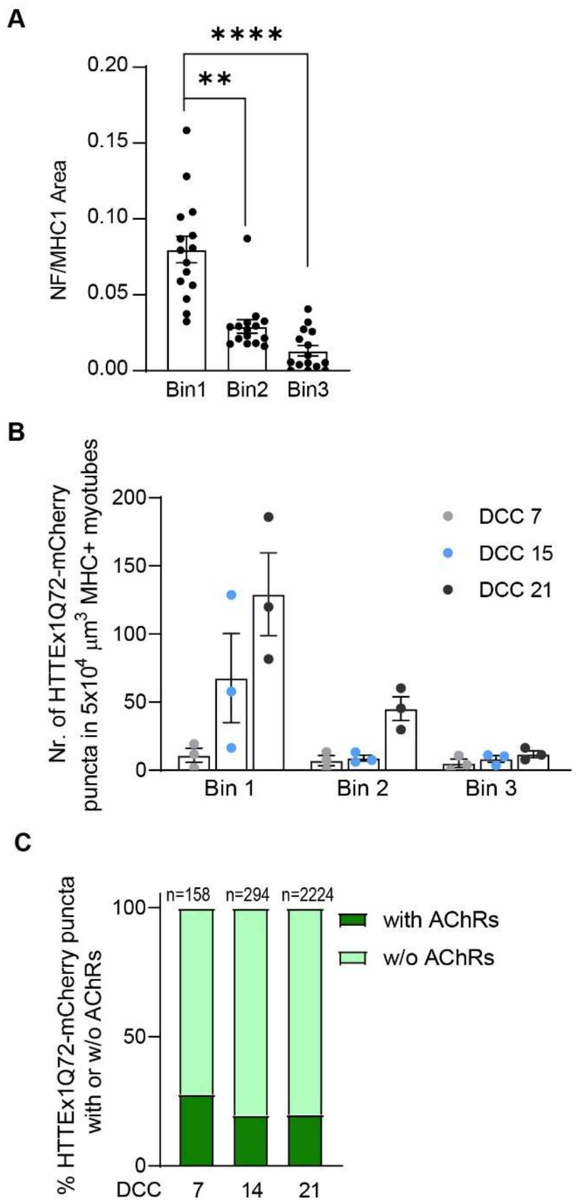

Supplemental Figure 9. HTTEx1Q72-mCherry neuromuscular transmission increases with co-culture time and with increasing neurofilament/myotube area. **(A)** Quantification of the NFM area normalized to the total MHC1 area in each bin in MFD at DCC 21. ** = $p \leq 0.01$; **** = $p < 0.0001$ (Kurskal-Wallis Test with Dunn's correction, n=15 pictures per bin from 1 experimental set). (**B**) Number of HTTEx1Q72-mCherry puncta in myotubes counted at DCC 7 – 21, in bin 1, 2, and 3 in the myotube compartment of a MFD (n=3 independent co-cultures/time point, data point: mean of 5 images). (**C**) Percentage of HTTEx1Q72-mCherry aggregates associated with AChR clusters at DCC 7 – 21. (n above each bar indicates the total number of aggregates analyzed). Abbreviation: **AChRs** = acetylcholine receptors; **DCC** = days of co-culture; **MHC** = myosin heavy chain, **NFM** = neurofilament m; **MFD**= microfluid devices. All averaged data are shown as the mean ± SEM.

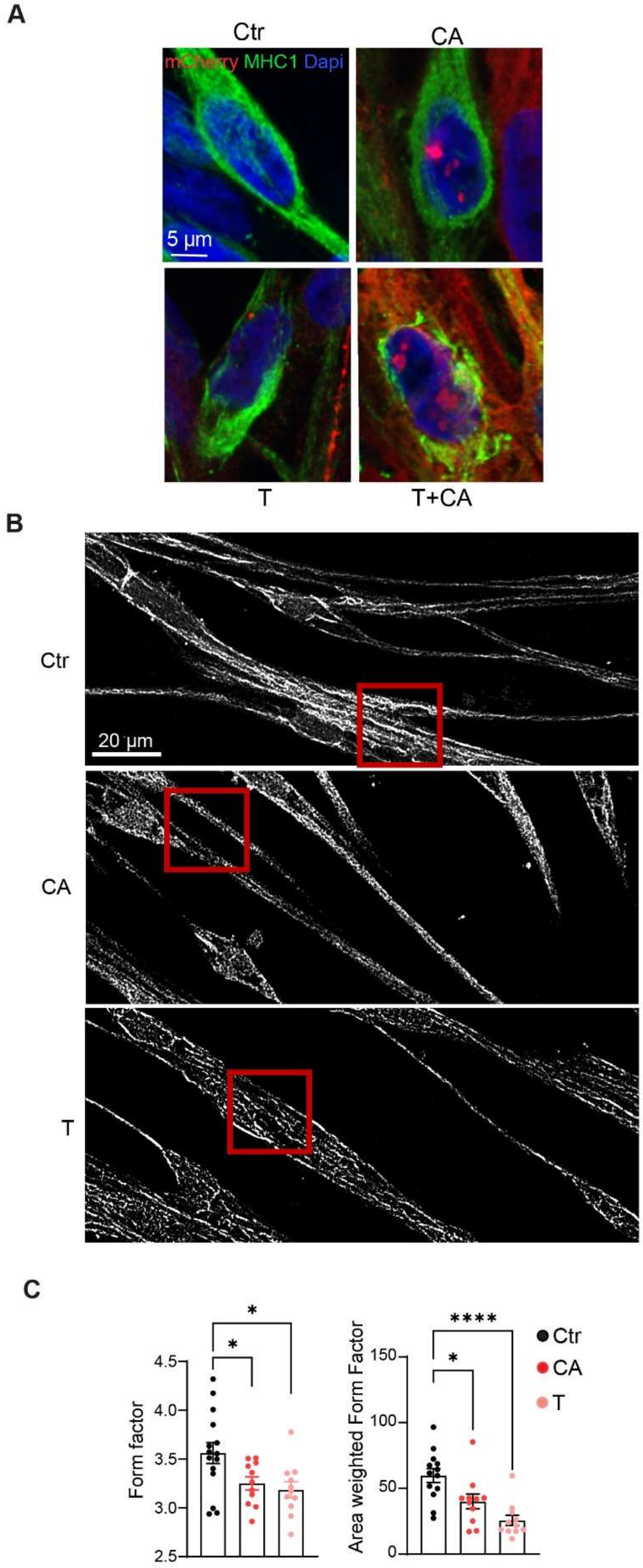

Supplementary Figure 10. Intranuclear HTTEx1Q72-mCherry aggregates and alterations of mitochondria structural parameters in myotubes upon transmission of HTTEx1Q72-mCherry from neurons. **(A)** An example of IF-labeled mixed-genotype neuromuscular co-cultures visualizing myotubes (MHC1) and their nucleus (Dapi) without or with HTTEx1Q72-mCherry. The quantification of the number of myotube nuclei with intranuclear HTTEx1Q72-mCherry aggregates (Figure 6D) was done using this labeling of myotubes with MHC1. **(B)** Representative microscopy images of the mitochondrial network morphology in myotubes. The TOMM20 mitochondrial marker is displayed in grey on the images. Red squares represent the cropped area presented in Figure 6E. **(C)** Graphs show the quantification of structural parameters of the mitochondria in the mixed-genotype co-cultures. (n=30-40 images/genotype from 3 independent MFD co-cultures). Abbreviations: **Ctr** = control; **CA** = cell autonomous; **T** = transmission; **T + CA** = transmission + cell autonomous.
