# Supplementary material for: Transmission-selective muscle pathology induced by the active propagation of mutant huntingtin across the human neuromuscular synapse
Source: Front Mol Neurosci. 2024 Jan 3;16:1287510. doi: 10.3389/fnmol.2023.1287510 (PMC10791992; doi:10.3389/fnmol.2023.1287510)
Supplement: Supplementary file 2 [file Data_Sheet_2.PDF]

Supplementary Table 1. Summary of statistical analysis for relevant figures.

Figure	Panel	Statistical Tests	Statistical Results
1	D	Two-way ANOVA	Interaction: F(2,69)=16.28, p<0.0001<br>Row Factor: F(2,69)=13.76, p<0.0001<br>Column Factor: F(1,69)=86.51, p<0.0001
1	E left	One-way ANOVA with post hoc tests	F(4,46)=16.69, p<0.0001<br>Tukey's multiple comparison tests:<br>DCC8 vs. 22: adj.p<0.0001<br>DCC15 vs. 29: adj.p=0.0019
1	E right	One-way ANOVA with post hoc tests	F(4,46)=10.95, p<0.0001<br>Tukey's multiple comparison tests:<br>DCC8 vs. 22: adj.p=0.0082<br>DCC15 vs. 22: adj.p=0.0486
1	G	Mixed-effects model (REML)	F (0.9122, 10.72) = 63.30, p<0.0001<br>Tukey's multiple comparison tests:<br>DCC4 vs. 7: adj.p=0.0017<br>DCC7 vs. 14: adj.p=0.0008<br>DCC14 vs. 21: adj.p=0.0144
2	D	Two-way ANOVA	F(2,7)=16.28, p<0.0001
2	E	One-way ANOVA with post hoc tests	F(5,77)=307.1, p<0.0001<br>Tukey's multiple comparison tests:<br>AMPA vs. AMPA+BgTx: adj.p<0.0001<br>AMPA vs. AMPA+TeNT: adj.p<0.0001<br>AMPA+BgTx vs. AMPA+TeNT, adj.p=0.9530<br>Glu vs. Glu+BgTx, adj.p<0.0001<br>Glu vs. Glu + TeNT, adj.p<0.0001<br>Glu+BgTx vs. Glu+TeNT, p=0.4246
2	G	One-way ANOVA with post hoc tests	F(2,6)=14.94, p<0.0047<br>Tukey's multiple comparison tests:<br>DCC7 vs. 21: adj.p=0.0042<br>DCC14 vs. 21: adj.p=0.0244
2	H	Kruskal-Wallis rank sum test, two-sided and pairwise Wilcoxon tests, two-sided	Kurskal-Wallis: p= 2.708e-10<br>Pairwise Wilcoxon with Benjamini-Hochberg multiple testing correction:<br>DCC7 vs. 15: adj.p= 9.7e-08<br>DCC15 vs. 21: adj.p= 1.9e-09
3	B	One-way ANOVA with post hoc tests	F(2,6)=30.78 p=0.0007<br>Tukey's multiple comparison tests:<br>bin1 vs. bin2: adj.p=0.003
3	C	One-way ANOVA with post hoc tests	F(2,6)=10.84 p=0.01<br>Tukey's multiple comparison tests:<br>bin1 vs. bin2: adj.p=0.04
3	E	Simple Linear Regression	R-squared=0.37<br>F(1,18)=10.42, p=0.0047
3	F	Fisher’s exact test two sided for DCC7 and 15, Chi-squared test for CCD21	DCC7 Fisher’s exact test: p=7.214e-09<br>DCC15: Fisher’s exact test: p<2.2e-16<br>DCC21: X-squared=674.03, df=3, p<2.2e-16

4	B	Unpaired t-test, two-sided	t=4.975, df=4, p=0.0076
4	C	Chi-squared test	X-squared=0.2981, df=3, p=0.9604
4	D	Unpaired t-test, two-sided	t=3.342, df=6, p=0.0156
4	E	Unpaired t-test, two-sided	t=2.604, df=6, p=0.0404
4	F	Unpaired t-test, two-sided	t=2.770, df=236, p=0.0061
4	G	Unpaired t-test, two-sided	t=0.6500, df=4, p=0.5511
4	H	One-way ANOVA with post hoc tests	F(3,8)=7.40, p=0.01<br>Tukey's multiple comparison tests:<br>mCherry Ctr vs. mCherry+TeNT: adj.p=0.9998<br>mCherry Ctr vs. Q72 Ctr: adj.p=0.0501<br>mCherry+TeNT vs. Q72+TeNT: adj.p=0.0351<br>Q72 Ctr vs. Q72+TeNT: adj.p = 0.9978
5	A	Unpaired t-test, two-sided	t=3.983, df=4, p=0.0164
5	B	Chi-squared test	X-squared =1.361, df=3, p=0.7147
5	C	Paired t-test, two- sided	t=10.27, df=32, p<0.0001
5	E	Unpaired t-test, two-sided	t=3.212, df=6, p=0.0183
5	F	Mann-Whitney test, two-sided	P = 0.0008
6	D	One-way ANOVA with post hoc tests	F (2, 25) = 25.22, p<0.0001<br>Tukey's multiple comparison tests:<br>CA vs. CA+T: adj.p=0.0003<br>T vs. CA+T: adj.p<0.0001
6	E Length	One-way ANOVA with post hoc tests	F(2, 34)=10.08, p=0.0004<br>Dunnett's multiple comparison tests:<br>Ctr vs CA: adj.p=0.0042<br>Ctr vs. T: adj.p=0.0004
6	F left panel	One-way ANOVA with post hoc tests	F(49, 744)=68.605, p<0.0001<br>Šidák’s multiple comparison test:<br>Ctr vs. T DCC8: adj.p=<0.0001<br>Ctr vs. T+CA DCC8:adj.p. <0.0001<br>Ctr vs. CA DCC15:adj.p. =0.0136<br>Ctr vs. T DCC15:adj.p. <0.0001<br>Ctr vs. T+CA DCC15:adj.p. <0.0001<br>Ctr vs. CA DCC22:adj.p. =0.0002<br>Ctr vs. T DCC22:adj.p. <0.0001<br>Ctr vs. T+CA DCC22:adj.p. <0.0001<br>Ctr vs. T DCC29:adj.p. <0.0001<br>Ctr vs. T+CA DCC29:adj.p. <0.0001
6	F right panel	One-way ANOVA with post hoc tests	F(49, 744)=66.87, p<0.0001<br>Šidák’s multiple comparison test:<br>Ctr vs. T DCC22:adj.p. <0.0001<br>Ctr vs. T+CA DCC22:adj.p. <0.0001

			Ctr vs. T DCC29:adj.p. <0.0001<br>Ctr vs. T+CA DCC29:adj.p. <0.0001
